# Dissemination patterns of Cochrane reviews on nutrition and physical activity using Altmetric data: a bibliographic study

**DOI:** 10.1186/s13643-026-03127-8

**Published:** 2026-02-23

**Authors:** Karina Karolina De Santis, Stefanie Maria Helmer, Katja Matthias

**Affiliations:** 1 Department of Prevention and Evaluation, Leibniz Institute for Prevention Research and Epidemiology—BIPS, Bremen, Germany; 2 Working Group Evidence-Based Public Health, Institute of Public Health and Nursing Research, University of Bremen, Bremen, Germany; 3 Faculty of Health Service, Catholic University of Applied Sciences of North Rhine-Westphalia, Wörthstraße 10, 50668 Cologne, Germany

**Keywords:** Cochrane review, Nutrition and physical activity, Altmetric Attention Score, Dimensions Badge, Dissemination

## Abstract

**Background:**

Cochrane reviews on nutrition and physical activity (PA) are highly relevant to scientists, practitioners, and the general public. It is important to investigate the dissemination patterns of these reviews to improve the uptake of their findings in practice. The aims of this study were (1) to describe the online and scientific attention towards Cochrane reviews on nutrition and PA using Altmetric data and (2) to investigate the associations between Altmetric data and review characteristics.

**Methods:**

This cross-sectional, bibliographic study used data from 249 Cochrane reviews on nutrition and PA for healthy or at-risk populations that were published in the Cochrane Database of Systematic Reviews from inception until May 2024. Review characteristics and open-access Altmetric data were extracted and analysed using relative frequencies. The associations among Altmetric Attention Scores (a measure of online attention), Dimensions Citations Scores (a measure of scientific attention), and review characteristics were computed using binary logistic regression analysis.

**Results:**

The 249 Cochrane reviews were published over a period of 25 years (1999–2024), included 0–195 studies, conducted a meta-analysis (80%), and had a plain language summary (PLS) available in 2–17 languages. Dissemination occurred via social media, including predominantly Twitter/X (*n* = 242), Facebook (*n* = 210), and Wikipedia (*n* = 172). Online attention based on the Altmetric Attention Scores ranged between 3 and 4111 (51% of reviews had scores between 3 and 48). Scientific attention based on the Dimensions Citations Scores ranged between 0 and 2700 (50% of reviews had scores between 0 and 81). Higher online attention was associated with higher scientific attention (odds ratio, OR = 11.14, 95% confidence interval, CI: 5.08–24.40) and more PLS languages (OR = 1.38, CI: 1.22–1.57). Higher scientific attention was associated with higher online attention (OR = 11.06, CI: 4.96–24.64), older publication year (OR = 0.12, CI: 0.06–0.27), more studies included in the review (OR = 4.13, CI: 1.90–8.98), and a meta-analysis conducted in the review (OR = 6.32, CI: 2.42–16.53).

**Conclusions:**

There was generally high online and scientific attention towards Cochrane reviews on nutrition and PA, mainly due to mentions in the social media and academic citations. Future studies need to investigate if higher online and scientific attention could enhance evidence uptake from Cochrane reviews in practice.

**Supplementary Information:**

The online version contains supplementary material available at 10.1186/s13643-026-03127-8.

## Background

Insufficient physical activity (PA) and poor dietary habits are major preventable risk factors for non-communicable diseases such as obesity, cardiovascular diseases, and diabetes [1, 2]. These factors have been identified by the World Health Organization (WHO) as key areas for action until 2030 [3].

The Cochrane Collaboration, known for its high standards in conducting systematic evidence syntheses, has recognised the need for high-quality evidence targeting specific health topics, leading to the establishment of the so-called Thematic Groups [4]. One such Thematic Group (‘Cochrane Nutrition and Physical Activity’ [5]) aims to support evidence-informed decision-making in policy and practice by producing and disseminating high-quality evidence syntheses on these topics [6]. The group especially highlights the need to make knowledge accessible and usable in practice by involving stakeholders in the production of evidence syntheses and by providing support for disseminating such evidence [6].

The appropriate dissemination of scientific findings is essential to ensure their uptake and application in real-world settings [7]. Dissemination involves sharing scientific knowledge strategically with targeted audiences through various channels [8]. Traditionally, dissemination relied on measuring the academic impact of a scientific publication via its citations in academic literature and its publication in a high-impact-factor journal [9]. However, due to technological advancements, open-access publishing, and the popularity of social media, dissemination strategies have shifted from targeting the scientific audience to reaching out to broader audiences and focusing on the social impact of academic publications and evidence uptake outside the scientific community [9–11]. Dissemination can be quantified using alternative metrics (or altmetrics) that measure the impact of scientific publications beyond their citations data. One source of such data is Altmetrics.com that belongs to the Digital Science portfolio of companies [12]. Altmetric data trace the scientific and social impact of academic publications. This is done through a weighted count of online mentions (Altmetric Attention Score [13]) to measure online attention and a count of citations (Dimensions Badge [14]) to measure scientific attention. Altmetric data suggest that Cochrane reviews in various health fields are associated with high scientific and online attention [15–18]. To support the further development and goals of the Thematic Group ‘Cochrane Nutrition and Physical Activity’, it is necessary to analyse the dissemination patterns of Cochrane reviews that have been published specifically on these topics so far and to identify the factors (i.e. review characteristics) that are associated with dissemination in these fields. Understanding such dissemination patterns can help to optimise efforts to ensure that high-quality evidence reaches the relevant stakeholders and the general public. In the fields of nutrition and PA, the appropriate dissemination of evidence from Cochrane reviews could raise awareness of the role of diet and exercise in promoting population health, which is the focus of the global call for action by the WHO [3]. Thus, this study investigated the dissemination patterns of Cochrane reviews on nutrition and PA. The aims of this study were (1) to describe the online and scientific attention towards these reviews using Altmetric data and (2) to investigate the associations between Altmetric data and review characteristics.

## Methods

### Study design

This is a bibliographic (i.e. research on research) study of open-access data on Cochrane reviews. The study uses a cross-sectional design and is embedded in an ongoing scoping review with a prospectively registered protocol [19]. Unlike planned in the protocol [19], all data were extracted and processed by human researchers and artificial intelligence tools were not used in any aspect of this study. The study adheres to the Strengthening the Reporting of Observational Studies in Epidemiology (STROBE) guideline [20]. The STROBE checklist is reported in Additional file 1.

### Data sources

The data source for this study was Cochrane reviews (i.e. systematic reviews of primary studies and overviews of reviews) published until May 2024. The inclusion criteria were (1) human populations of any age or sex (either healthy, at risk for clinical conditions, or mixed populations, including healthy, at risk, and people with clinical conditions) and (2) any intervention or concept targeting nutrition and PA in the context of health promotion and disease prevention. The exclusion criteria were (1) clinical or special populations (e.g. premature babies) undergoing clinical treatment in healthcare settings, (2) interventions or concepts targeting fields other than nutrition or PA, and (3) other Cochrane publications (e.g. review protocols or older reviews with published updates that were already included in this study).

The Cochrane reviews were located in the Cochrane Database of Systematic Reviews (from inception until May 2024). One researcher (KM) developed, calibrated, and pilot-tested the search syntax using feedback from the team, developed the final search strategy (Additional file 2), conducted the electronic search on 14 May 2024, and stored the results in EndNote 20 (Clarivate). Review selection was done independently by two researchers (KM and SMH) using title and abstract, and full-text screening in Rayyan (Rayyan Systems, USA). Any disagreements were resolved by discussion.

### Data collection process

Data were manually extracted from the full texts of the reviews, the review information on the Cochrane website, and the Altmetric data linked to each review on the Cochrane website. The data extraction sheet was developed in Excel 10 (Microsoft Inc.) by the study authors, pilot-tested and calibrated within the team. All researchers extracted different aspects of the data. To reduce bias in data extraction, all data were subsequently cross-checked by one researcher (KKDS), and any discrepancies were resolved by discussion with another researcher (KM).

### Variables and data processing

The variables used in this study included review characteristics and Altmetric data of 249 Cochrane reviews on nutrition and PA (Table 1).

**Table 1:** Variables used in this study

Variable	Variable coding	Data source
Review characteristics		
Review type	Systematic, overview of reviews	Review full-text
Review publication year	Year	Review full-text
Studies included in the review	Number	Review full-text
Meta-analysis conducted in the review	Yes or no	Review full-text
Plain language summary (PLS) languages^a^	Number and list of languages	Cochrane website
Altmetric data^a^		
Altmetric Attention Score (online attention)	Number	Cochrane website
Altmetric Attention Score interpretation	In the top 5% of all research outputs scored by Altmetric: yes or no	Cochrane website
Altmetric Attention Score channels that mentioned the Cochrane review at least once	Number and list of channels	Cochrane website
Dimensions Citations Score (scientific attention)	Number	Cochrane website

Data extracted for the dynamic variables (marked with ^a^), which are updated every 24 h, were cross-checked with the Cochrane website on 25 October 2024 by one researcher (KKDS)

Altmetric data are partially available open-access and can be used to objectively quantify and explore the online dissemination of academic publications, such as Cochrane reviews. Two Altmetric measures were used in this study: (1) the Altmetric Attention Score and (2) the Dimensions Badge (Table 1). The Altmetric Attention Score is a measure of online attention towards academic publications based on their mentions in various online channels (e.g. Wikipedia, YouTube, and Twitter/X) weighted by the channel type [21]. The Altmetric Attention Score interpretation is reported together with each Altmetric Attention Score. It is a measure that compares each Altmetric Attention Score relative to scores of similar sources traced by Altmetric, based on age (i.e. with the same publication year) and outlet (e.g. in the same scientific journal). The Attention Score interpretation is reported as a percentage score using categories defined by Altmetric (e.g. ‘Altmetric Attention Score in the top 5% of all research outputs scored by Altmetric’). The Dimensions Badge (that is also referred to as the Dimensions Citations Score) shows the total number of citations of an academic publication in the outlets such as scientific journals indexed by the Dimensions database [14]. The Dimensions database is comparable to other international bibliographic databases based on a high overlap in the coverage of over one million citations in health and medical sciences with SCOPUS (86%) and Web of Science (89%) [22].

In this study, the Altmetric Attention Score was used as a measure of online attention and the Dimensions Citations Score as a measure of scientific attention towards each Cochrane review.

### Data analysis

The distribution of scores for each variable was assessed using frequency analysis and box plots in Excel and IBM SPSS Statistics 29. Continuous variables with skewed distributions and outliers were dichotomised using the median. The median value was always included in the lower-coded category.

The data were analysed using descriptive statistics (i.e. absolute and relative frequencies, medians and ranges). Associations between each attention type (i.e. online and scientific attention) and review characteristics were computed using binary logistic regression analysis. Multicollinearity was tested using bivariate correlation coefficients between any two independent variables and goodness-of-fit was measured by Hosmer and Lemeshow test.

## Results

### Study selection

Among 1912 sources identified in the electronic search, 1620 were excluded in the title and abstract screening due to fulfilling at least one exclusion criterion and 43 were excluded in the full-text screening based on exclusion criterion 1 (clinical populations; *n* = 24), exclusion criterion 2 (no focus on nutrition or PA; *n* = 17), or exclusion criterion 3 (other Cochrane publications; *n* = 2). The remaining 249 Cochrane reviews were included in this study (Additional file 2). All reviews included human populations of any age or sex focusing on healthy or at-risk groups and interventions or concepts targeting nutrition and PA in the context of health promotion and disease prevention.

### Characteristics of the included studies (249 Cochrane reviews)

**Fig. 1:**
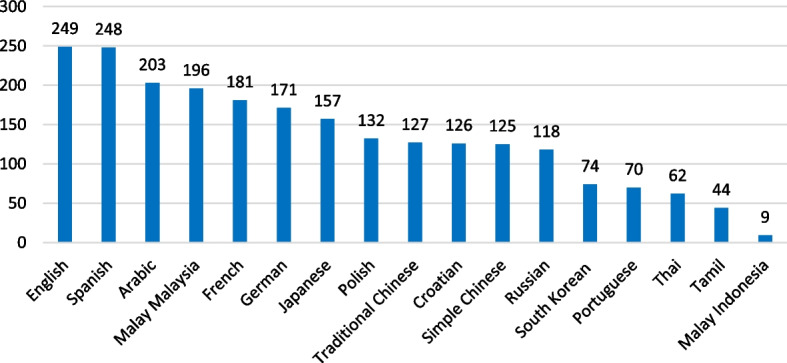
Plain language summary (PLS) languages of the included 249 Cochrane reviews

This study includes data from 249 Cochrane reviews (*n* = 245 systematic and *n* = 4 overviews of reviews). The reviews were published over a period of 25 years (1999–2024), included 0–195 studies (0 studies in *n* = 7 reviews and 1–195 studies in *n* = 242 reviews), conducted a meta-analysis (80%), and had a plain language summary (PLS) available in 2–17 languages (Table 2). Most common languages were English (*n* = 249), Spanish (*n* = 248), and Arabic (*n* = 203; Fig. 1).

**Table 2:** Characteristics of the included studies (249 Cochrane reviews)

Variable	Range	Median if skewed distribution	Categories	*n* (% of 249)
Publication year	1999–2024	2016	Older (1999–2016)	136 (55%)
			Newer (2017–2024)	113 (45%)
Studies included in the review^a^	0–195	13	Less (0–13)	125 (50%)
			More (14–195)	124 (50%)
Meta-analysis conducted in the review	–	–	Yes	198 (80%)
			No	51 (20%)
Plain language summary (PLS) languages	2–17	–	–	–
Altmetric Attention Score (online attention)^a^	3–4111	48	Lower (3–48)	126 (51%)
			Higher (49–4111)	123 (49%)
Altmetric Attention Score interpretation (review in the top 5% of all traced outputs)	–	–	Yes	148 (59%)
			No	101 (41%)
Dimensions Citations Score (scientific attention)^a^	0–2700	81	Lower (0–81)	125 (50%)
			Higher (82–2700)	124 (50%)

Outliers were identified using box plots (Additional file 3, Outliers). ^a^Variables with skewed distributions and outliers were dichotomised using the median (Additional file 3, Medians). The median value was always included in the lower-coded category

### Online and scientific attention towards 249 Cochrane reviews

The Altmetric Attention Score (online attention) ranged between 3 and 4111 and 51% of reviews had scores between 3 and 48 (Table 2). Online attention was high relative to other sources traced by Altmetric because 59% of the included Cochrane reviews were rated within the top 5% of all traced research outputs (Table 2). The Cochrane review with the highest Altmetric Attention Score of 4111 was published in 2013 and investigated the nutritional supplementation with vitamin C for preventing and treating a common cold [23].

The Dimensions Citations Scores (scientific attention) ranged between 0 and 2700 and 50% of reviews had scores between 0 and 81 that indicate the total number of citations of each Cochrane review in the sources indexed by Dimensions (Table 2). The Cochrane review with the highest Dimensions Citations Score of 2700 was published in 2012 and investigated the interventions for preventing falls in older people living in the community [24].

**Fig. 2:**
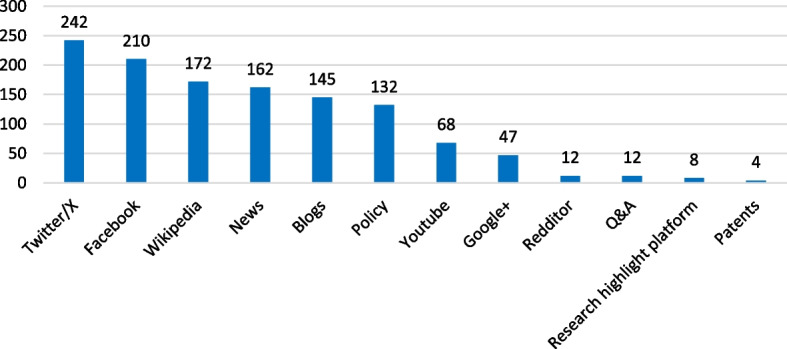
Online channels traced by Altmetric that mentioned the included Cochrane reviews at least once

Several online channels traced by Altmetric mentioned the included Cochrane reviews at least once. These were predominantly social media channels, including Twitter/X (*n* = 242), Facebook (*n* = 210), and Wikipedia (*n* = 172; Fig. 2).

### Review characteristics associated with online and scientific attention

The associations between review characteristics and online and scientific attention were computed using two binary logistic regression analyses (Table 3). Since some continuous variables (dependent and independent) had skewed distributions and outliers, a sensitivity analysis was also computed using two linear regression analyses with ranked variables (Additional file 3, Sensitivity analysis 1 and 2).

**Table 3:** Review characteristics associated with online and scientific attention towards 249 Cochrane reviews

Independent variable (review characteristics)	Independent variable coding	Analysis 1: online attention (odds ratio [95% confidence interval])	Analysis 2: scientific attention (odds ratio [95% confidence interval])
(1) Publication year	Nominal: newer (2017–2024) vs. older (1999–2016) (R)	*1.17 [0.55–2.48]*^*a*^	0.12 [0.06–0.27]*
(2) Studies included in the review	Nominal: more (14–195) vs. less (0–13) (R)	1.67 [0.85–3.28]	4.13 [1.90–8.98]*
(3) Meta-analysis conducted in the review	Nominal: yes vs. no (R)	0.70 [0.30–1.64]	6.32 [2.42–16.53]*
(4) Plain language summary (PLS) languages	Scale: 2–17	1.38 [1.22–1.57]*	0.84 [0.74–0.96]*^b^
(5) Online attention (Altmetric Attention Score)	Nominal: higher (49–4111) vs. lower (3–48) (R)	–	11.06 [4.96–24.64]*
(6) Scientific attention (Dimensions Citations Score)	Nominal: higher (82–2700) vs. lower (0–81) (R)	11.14 [5.08–24.40]*	–

Results of two binary logistic regression analyses. Reference categories (R) were always coded as 0. Multicollinearity was absent based on bivariate correlation coefficients between any two independent variables of less than ±0.4 (Additional file 3, Multicollinearity). Analysis 1: dependent variable (Altmetric Attention Scores) was coded as 0 = lower (3–48) and 1 = higher (49–4111) scores; overall model: *Χ*^2^ = 93.55, df = 5, *p* < 0.001, Cox and Snell *R*^2^ = 31%; model goodness-of-fit assumed (Hosmer and Lemeshow test: *Χ*^2^ = 5.61, df = 8, *p* = 0.691). Analysis 2: dependent variable (Dimensions Citations Scores) was coded as 0 = lower (0–81) and 1 = higher (82–2700); overall model: *Χ*^2^ = 125.78, df = 5, *p* < 0.001, Cox and Snell *R*^2^ = 40%; model goodness-of-fit assumed (Hosmer and Lemeshow test: *Χ*^2^ = 6.72, df = 8, *p* = 0.567)

The associations from the binary logistic regressions were confirmed in terms of direction and significance in the two sensitivity analyses with two exceptions: ^a^higher online attention was also significantly associated with newer publication year (sensitivity analysis 1) and ^b^scientific attention was not associated with PLS languages (sensitivity analysis 2; Additional file 3)

*p < 0.05

Higher online attention was associated with higher scientific attention and more PLS languages in the main analysis (Table 3, Analysis 1) and in the sensitivity analysis (Additional file 3, Sensitivity analysis 1). In addition, the sensitivity analysis showed that higher online attention was associated with newer publication year (Additional file 3, Sensitivity analysis 1).

Higher scientific attention was associated with higher online attention, older publication year, more studies included in the review, and a meta-analysis conducted in the review in the main analysis (Table 3, Analysis 2) and in the sensitivity analysis (Additional file 3, Sensitivity analysis 2). Sensitivity analysis showed that scientific attention was not associated with PLS languages (Additional file 3, Sensitivity analysis 2).

### Dissemination of this study

In addition to this academic publication, the results of this study were disseminated as a presentation at a scientific meeting [25] and PLS in English and German (Additional file 4).

## Discussion

### Summary of main findings

This bibliographic study shows that the online and scientific attention towards 249 Cochrane reviews on nutrition and PA was in general high based on Altmetric data. The dissemination of Cochrane reviews occurred predominantly through mentions in the social media, including Twitter/X, Facebook, and Wikipedia, and through scientific citations. Online and scientific attention were positively associated with each other and with some review characteristics. In general, attention towards the 249 Cochrane reviews increased over time since publication and was higher for reviews with more data (i.e. a higher number of included studies), reviews with a meta-analysis, and reviews that could reach a more international audience (i.e. those with PLS available in more languages).

### Dissemination

Since online attention towards Cochrane reviews on nutrition and PA, as well as in other health fields [15–18], is high, such reviews continue to reach a wide online audience. However, it is unclear who is responsible for creating online content on these reviews in the social media relative to the traceable authors of scientific citations (e.g. the Cochrane review authors themselves or other researchers). Research shows that publications with company participation receive more online attention [26]. Indeed, one important and traceable contributor to the online attention towards Cochrane reviews is Cochrane itself because, as an institution, it actively promotes its content through social media, including Twitter/X and Wikipedia [18]. The motivation to create online content on scientific publications, including Cochrane reviews, is otherwise unclear. The online content on Cochrane reviews can be created by anyone for legitimate purposes, such as to connect the scientific community with the general public, to educate and provide access to health information, and to foster the active debate on health issues among the general public [11]. However, there are several risks with the uptake of such content, including the potential manipulation and promotion of health misinformation by content creators, difficulties with understanding of complex health topics by both the content creators and the general public, and, as a consequence, an increase in public distrust in scientific research [11]. If dissemination is done by health professionals and researchers, then institutional policies, training, and guidance are needed to reduce such risks [11]. More research is needed to identify effective measures to prevent the spread of health misinformation by private content creators online.

The bivariate correlations between Altmetric Attention Scores and citation counts are positive but typically only weak to moderate for Cochrane reviews and other publications in health fields [9, 10, 15, 27, 28]. Online attention may not always parallel the traditional citation metrics for several reasons. First, as satirically pointed out in the context of tracing attention towards humans using Altmetric data [29, 30], both measures of attention address different constructs, including fame rather than scientific merit of researchers. Thus, any association between online and scientific attention towards academic publications could also be spurious or, at best, only weak. Second, attention depends on the topic. Scientific publications addressing more applied or broader health topics, such as nutrition and PA, are more relevant for the general public and may receive higher online attention relative to other specific health topics that may be less relevant beyond the scientific community [9, 10]. Third, online attention can increase faster (e.g. via mentions in the social media immediately after review publication) relative to slower increase in scientific attention (i.e. longer time needed for the academic publications to be cited) [9, 27]. Fourth, the reliability and validity of Altmetric data have been criticised due to narrow channel coverage and limited transparency in score computation [31]. These issues likely bias the correlation between online and scientific attention because some academic publications may receive high online attention due to negative reception, they may be undetected online due to incorrect indexing (e.g. if they have an incorrect or no doi, or are not included in academic databases traced by Dimensions), and their scores could be affected if accounts in specific online channels are closed [31]. Thus, to provide a more accurate measure of attention towards Cochrane reviews and other scientific publications, Altmetric data would need to include a wider range of international online media channels to cover regionally and linguistically diverse scientific content and transparent reporting on how the scores are computed to improve their interpretation [31]. Due to the limitations of Altmetric data, it is essential to consider such data as complementary tools, rather than a replacement for traditional citation-based metrics [10]. It is also important to consider that higher online attention is not associated with higher quality of evidence reported in review [7], nor with other proxies for evidence quality, including the number of studies in review and conduct of a meta-analysis (Table 2). However, as a complementary tool, Altmetric data could be used to identify studies that have attracted high attention in a specific field to subsequently consider them for use in practice (e.g. for clinical decision-making) [32] pending their critical appraisal and risk of bias assessment.

One interesting topic that was not investigated in this study is the potential causality between online and scientific attention towards Cochrane reviews. This study shows that both types of attention are strongly and positively associated when other review characteristics are controlled for in the regression analyses (Table 3). Therefore, it can be speculated that higher online attention is driven by higher scientific attention and more PLS languages. This is likely as scientific publications could be discussed online (e.g. mentioned in news coverage or linked to Wikipedia content) and reach more international audiences if their PLS is available in more languages. The opposite direction of causation could also be true in that higher scientific attention may be driven by higher online attention and the specific bibliographic characteristics of reviews. Although less plausible, the scientific attention could increase because researchers may become aware of the new scientific publications in the online channels traced by Altmetric and subsequently cite these publications in their own scientific output. Furthermore, as logically expected, the scientific attention also appears to be driven by the bibliographic characteristics of reviews. Higher scientific attention was observed in reviews with more data (i.e. with more included studies), in reviews with a meta-analysis, and in older review with earlier publication dates. Since online attention fluctuates over time and depends on the traced online channel [15, 18], future studies need to examine how online and scientific attention interact over time. A better understanding of the relationship between online and scientific attention could help to further enhance the visibility and potential uptake of evidence from Cochrane reviews.

### Limitations

There were several limitations in this study. First, the Altmetric data for Cochrane reviews could be inflated due to the Cochrane strategy of disseminating its own content through social media. Thus, the high attention towards Cochrane reviews may not be generalisable to other non-Cochrane systematic reviews. Second, this study included a single group of Cochrane reviews focusing on nutrition and PA without a comparison group (e.g. Cochrane reviews in other health fields). Altmetric data show that more than half of these reviews received particularly high online attention scores compared to other Cochrane reviews of a similar age and source. Future research could investigate measures that could be taken to increase the attention towards Cochrane reviews on other, more specialised or basic health research topics. Third, this study did not examine the content of online mentions or scientific publications referring to Cochrane reviews, the authors of such content, nor any temporal data related to online attention (e.g. the time lag between the publication date of each Cochrane review and their online mentions, the total duration of display of online mentions, or any global trends in the use of various online channels). Future research is needed to better understand who mentions Cochrane reviews, why and when they do so, and how the mentions perform over time. It is also important to examine whether online mentions or scientific citations of Cochrane reviews facilitate evidence uptake from these reviews in practice. Fourth, this study included variables with skewed distributions and outliers in two separate statistical analyses. Further data on review content, including population and intervention types, the quality of the reported evidence, and review outcomes (e.g. if interventions were effective) could be used to compute a more complex multivariate model with two attention types as dependent variables. This could be done to determine how the interaction between the online and scientific attention is mediated or moderated by review content in addition to the review characteristics included in this study. Fifth, the content or quality of the evidence in the Cochrane reviews were not assessed in this study. The content of the included Cochrane reviews will be examined in an ongoing scoping review [19]. This will be done to identify evidence gaps in terms of population groups or intervention foci that were not included in the past Cochrane reviews on nutrition and PA [19]. This information could be used by the Thematic Group ‘Cochrane Nutrition and Physical Activity’ to identify priorities for future Cochrane reviews. Further research is also needed to identify measures that could increase online attention, particularly towards reviews with a high quality of evidence.

## Conclusions

There was generally high online and scientific attention towards Cochrane reviews on nutrition and PA, mainly due to mentions in the social media and academic citations. Future studies need to investigate if higher online and scientific attention could enhance evidence uptake from Cochrane reviews in practice.

## Supplementary Information

Supplementary Material 1. STROBE checklist.

Supplementary Material 2. Search strategy and list of included studies.

Supplementary Material 3. Sensitivity analysis.

Supplementary Material 4. Plain language summary.

## Data Availability

The dataset supporting the conclusions of this article can be requested from the corresponding author.

## Abbreviations

PA: Physical activity
PLS: Plain language summary
STROBE: Strengthening the Reporting of Observational Studies in Epidemiology
WHO: World Health Organization

## Declarations

Ethics approval and consent to participate

Not applicable.
