# Supplementary material for: Dissemination patterns of Cochrane reviews on nutrition and physical activity using Altmetric data: a bibliographic study
Source: Syst Rev. 2026 Feb 23;15:104. doi: 10.1186/s13643-026-03127-8 (PMC13037133; doi:10.1186/s13643-026-03127-8)
Supplement: Supplementary file 1 — Supplementary Material 1. STROBE checklist. [file 13643_2026_3127_MOESM1_ESM.docx]

**Appendix 1**

**STROBE Checklist**

| **Item** | | Item No | Recommendation | Location in the manuscript |
| --- | --- | --- | --- | --- |
| **Title and abstract** | | 1 | (*a*) Indicate the study’s design with a commonly used term in the title or the abstract | Title |
|  |  |  | (*b*) Provide in the abstract an informative and balanced summary of what was done and what was found | Abstract |
| Introduction | | | |  |
| Background/rationale | | 2 | Explain the scientific background and rationale for the investigation being reported | Introduction |
| Objectives | | 3 | State specific objectives, including any prespecified hypotheses | Introduction |
| Methods | | | |  |
| Study design | | 4 | Present key elements of study design early in the paper | Study design |
| Setting | | 5 | Describe the setting, locations, and relevant dates, including periods of recruitment, exposure, follow-up, and data collection | Data source |
| Participants | | 6 | (*a*) *Cross-sectional study*—Give the eligibility criteria, and the sources and methods of selection of participants | Data sources |
| Variables | | 7 | Clearly define all outcomes, exposures, predictors, potential confounders, and effect modifiers. Give diagnostic criteria, if applicable | Variables and data processing |
| Data sources/ measurement | | 8* | For each variable of interest, give sources of data and details of methods of assessment (measurement). Describe comparability of assessment methods if there is more than one group | Variables and data processing |
| Bias | | 9 | Describe any efforts to address potential sources of bias | Data collection process |
| Study size | | 10 | Explain how the study size was arrived at | Data sources |
| Quantitative variables | | 11 | Explain how quantitative variables were handled in the analyses. If applicable, describe which groupings were chosen and why | Data synthesis |
| Statistical methods | | 12 | (*a*) Describe all statistical methods, including those used to control for confounding | Data analysis |
|  |  |  | (*b*) Describe any methods used to examine subgroups and interactions | Data analysis |
|  |  |  | (*c*) Explain how missing data were addressed | N/A |
|  |  |  | (*d*) *Cross-sectional study*—If applicable, describe analytical methods taking account of sampling strategy | N/A |
|  |  |  | (*e*) Describe any sensitivity analyses | N/A |
| Results | | | |  |
| Participants | 13* | (a) Report numbers of individuals at each stage of study—eg numbers potentially eligible, examined for eligibility, confirmed eligible, included in the study, completing follow-up, and analysed | | Methods/ Data sources |
|  |  | (b) Give reasons for non-participation at each stage | | N/A |
|  |  | (c) Consider use of a flow diagram | | N/A |
| Descriptive data | 14* | (a) Give characteristics of study participants (eg demographic, clinical, social) and information on exposures and potential confounders | | Table 1, Figures 1-2 |
|  |  | (b) Indicate number of participants with missing data for each variable of interest | | N/A |
|  |  | (c) *Cohort study*—Summarise follow-up time (eg, average and total amount) | | N/A |
| Outcome data | 15* | *Cohort study*—Report numbers of outcome events or summary measures over time | | N/A |
|  |  | *Case-control study—*Report numbers in each exposure category, or summary measures of exposure | | N/A |
|  |  | *Cross-sectional study—*Report numbers of outcome events or summary measures | | Tables 2-3 |
| Main results | 16 | (*a*) Give unadjusted estimates and, if applicable, confounder-adjusted estimates and their precision (eg, 95% confidence interval). Make clear which confounders were adjusted for and why they were included | | Table 3 |
|  |  | (*b*) Report category boundaries when continuous variables were categorized | | N/A |
|  |  | (*c*) If relevant, consider translating estimates of relative risk into absolute risk for a meaningful time period | | N/A |
| Other analyses | 17 | Report other analyses done—eg analyses of subgroups and interactions, and sensitivity analyses | | N/A |
| Discussion | | | |  |
| Key results | 18 | Summarise key results with reference to study objectives | | Summary of main findings |
| Limitations | 19 | Discuss limitations of the study, taking into account sources of potential bias or imprecision. Discuss both direction and magnitude of any potential bias | | Limitations |
| Interpretation | 20 | Give a cautious overall interpretation of results considering objectives, limitations, multiplicity of analyses, results from similar studies, and other relevant evidence | | Discussion/ Dissemination |
| Generalisability | 21 | Discuss the generalisability (external validity) of the study results | | Limitations |
| Other information | | | |  |
| Funding | 22 | Give the source of funding and the role of the funders for the present study and, if applicable, for the original study on which the present article is based | | Funding |

Source: von Elm E, Altman DG, Egger M, Pocock SJ, Gøtzsche PC, Vandenbroucke JP. Strengthening the Reporting of Observational Studies in Epidemiology (STROBE) Statement: Guidelines for reporting observational studies. *BMJ*. 2007; 335(7624): 806. doi:10.1136/bmj.39335.541782.AD
