# Supplementary material for: Dissemination patterns of Cochrane reviews on nutrition and physical activity using Altmetric data: a bibliographic study
Source: Syst Rev. 2026 Feb 23;15:104. doi: 10.1186/s13643-026-03127-8 (PMC13037133; doi:10.1186/s13643-026-03127-8)
Supplement: Supplementary file 2 — Supplementary Material 2. Search strategy and list of included studies. [file 13643_2026_3127_MOESM2_ESM.docx]

**Appendix 2**

**Search syntax**


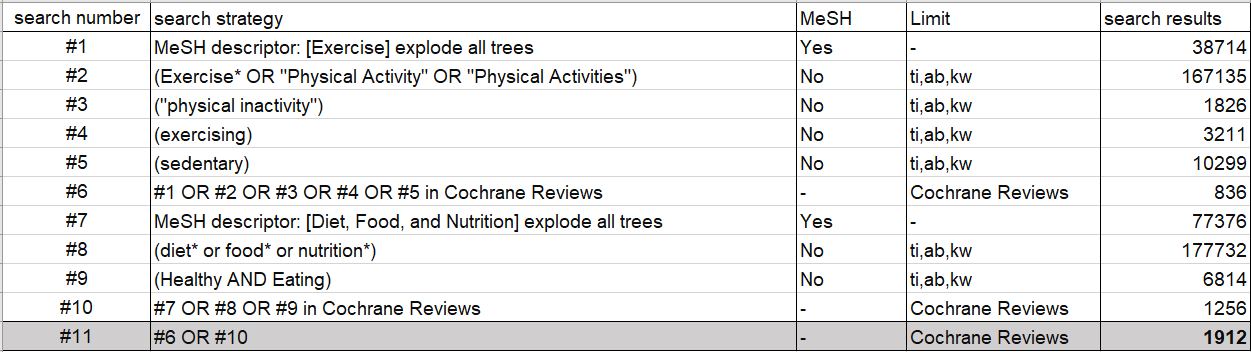


**List of included Cochrane reviews (n=249)**

1. Aas R, Tuntland H, Holte K, Røe C, Lund T, Marklund S, et al. Workplace interventions for neck pain in workers. Cochrane Database of Systematic Reviews. 2011 2011(4). doi: doi:10.1002/14651858.CD008160.pub2.
2. Abdelhamid AS, Brown TJ, Brainard JS, Biswas P, Thorpe GC, Moore HJ, et al. Omega‐3 fatty acids for the primary and secondary prevention of cardiovascular disease. Cochrane Database of Systematic Reviews. 2020 (3). doi: 10.1002/14651858.CD003177.pub5.
3. Abdelhamid AS, Martin N, Bridges C, Brainard JS, Wang X, Brown TJ, et al. Polyunsaturated fatty acids for the primary and secondary prevention of cardiovascular disease. Cochrane Database of Systematic Reviews. 2018 (11). doi: 10.1002/14651858.CD012345.pub3.
4. Abdulwadud O, Snow M. Interventions in the workplace to support breastfeeding for women in employment. Cochrane Database of Systematic Reviews. 2012 2012(10). doi: doi:10.1002/14651858.CD006177.pub3.
5. Abe SK, Balogun OO, Ota E, Takahashi K, Mori R. Supplementation with multiple micronutrients for breastfeeding women for improving outcomes for the mother and baby. Cochrane Database of Systematic Reviews. 2016 (2). doi: 10.1002/14651858.CD010647.pub2.
6. Adler AJ, Taylor F, Martin N, Gottlieb S, Taylor RS, Ebrahim S. Reduced dietary salt for the prevention of cardiovascular disease. Cochrane Database of Systematic Reviews. 2014 (12). doi: 10.1002/14651858.CD009217.pub3.
7. Al‐Khudairy L, Flowers N, Wheelhouse R, Ghannam O, Hartley L, Stranges S, et al. Vitamin C supplementation for the primary prevention of cardiovascular disease. Cochrane Database of Systematic Reviews. 2017 (3). doi: 10.1002/14651858.CD011114.pub2.
8. Al‐Khudairy L, Loveman E, Colquitt J, Mead E, Johnson R, Fraser H, et al. Diet, physical activity and behavioural interventions for the treatment of overweight or obese adolescents aged 12 to 17 years. Cochrane Database of Systematic Reviews. 2017 2017(6). doi: doi:10.1002/14651858.CD012691.
9. Amorim Adegboye AR, Linne YM. Diet or exercise, or both, for weight reduction in women after childbirth. Cochrane Database of Systematic Reviews. 2013 (7). doi: 10.1002/14651858.CD005627.pub3.
10. Arikpo D, Edet E, Chibuzor M, Odey F, Caldwell D. Educational interventions for improving primary caregiver complementary feeding practices for children aged 24 months and under. Cochrane Database of Systematic Reviews. 2018 2018(5). doi: doi:10.1002/14651858.CD011768.pub2.
11. Armanian A, Jahanfar S, Feizi A, Salehimehr N, Molaeinezhad M, Sadeghi E. Prebiotics for the prevention of hyperbilirubinaemia in neonates. Cochrane Database of Systematic Reviews. 2019 2019(8). doi: doi:10.1002/14651858.CD012731.pub2.
12. Ashworth NL, Chad KE, Harrison EL, Reeder BA, Marshall SC. Home versus center based physical activity programs in older adults. Cochrane Database of Systematic Reviews. 2005 (1). doi: 10.1002/14651858.CD004017.pub2.
13. Avenell A, Mak JCS, O'Connell DL. Vitamin D and vitamin D analogues for preventing fractures in post‐menopausal women and older men. Cochrane Database of Systematic Reviews. 2014 (4). doi: 10.1002/14651858.CD000227.pub4.
14. Azarpazhooh A, Lawrence H, Shah P. Xylitol for preventing acute otitis media in children up to 12 years of age. Cochrane Database of Systematic Reviews. 2016 2016(8). doi: doi:10.1002/14651858.CD007095.pub3.
15. Baker PRA, Francis DP, Soares J, Weightman AL, Foster C. Community wide interventions for increasing physical activity. Cochrane Database of Systematic Reviews. 2015 (1). doi: 10.1002/14651858.CD008366.pub3.
16. Baldwin C, Kimber K, Gibbs M, Weekes C. Supportive interventions for enhancing dietary intake in malnourished or nutritionally at‐risk adults. Cochrane Database of Systematic Reviews. 2016 2016(12). doi: doi:10.1002/14651858.CD009840.pub2.
17. Balogun O, da Silva Lopes K, Ota E, Takemoto Y, Rumbold A, Takegata M, et al. Vitamin supplementation for preventing miscarriage. Cochrane Database of Systematic Reviews. 2016 2016(5). doi: doi:10.1002/14651858.CD004073.pub4.
18. Balogun O, O'Sullivan E, McFadden A, Ota E, Gavine A, Garner C, et al. Interventions for promoting the initiation of breastfeeding. Cochrane Database of Systematic Reviews. 2016 2016(11). doi: doi:10.1002/14651858.CD001688.pub3.
19. Baxter JA, Carducci B, Kamali M, Zlotkin SH, Bhutta ZA. Fortification of salt with iron and iodine versus fortification of salt with iodine alone for improving iron and iodine status. Cochrane Database of Systematic Reviews. 2022 (4). doi: 10.1002/14651858.CD013463.pub2.
20. Bergwall S, Johansson A, Sonestedt E, Acosta S. High versus low‐added sugar consumption for the primary prevention of cardiovascular disease. Cochrane Database of Systematic Reviews. 2022 (1). doi: 10.1002/14651858.CD013320.pub2.
21. Bjelakovic G, Gluud LL, Nikolova D, Whitfield K, Krstic G, Wetterslev J, et al. Vitamin D supplementation for prevention of cancer in adults. Cochrane Database of Systematic Reviews. 2014 (6). doi: 10.1002/14651858.CD007469.pub2.
22. Bjelakovic G, Gluud LL, Nikolova D, Whitfield K, Wetterslev J, Simonetti RG, et al. Vitamin D supplementation for prevention of mortality in adults. Cochrane Database of Systematic Reviews. 2014 (1). doi: 10.1002/14651858.CD007470.pub3.
23. Bjelakovic G, Nikolova D, Simonetti RG, Gluud C. Antioxidant supplements for preventing gastrointestinal cancers. Cochrane Database of Systematic Reviews. 2008 (3). doi: 10.1002/14651858.CD004183.pub3.
24. Brand A, Visser ME, Schoonees A, Naude CE. Replacing salt with low‐sodium salt substitutes (LSSS) for cardiovascular health in adults, children and pregnant women. Cochrane Database of Systematic Reviews. 2022 (8). doi: 10.1002/14651858.CD015207.
25. Bricker L, Reed K, Wood L, Neilson JP. Nutritional advice for improving outcomes in multiple pregnancies. Cochrane Database of Systematic Reviews. 2015 (11). doi: 10.1002/14651858.CD008867.pub3.
26. Brown T, Moore THM, Hooper L, Gao Y, Zayegh A, Ijaz S, et al. Interventions for preventing obesity in children. Cochrane Database of Systematic Reviews. 2019 (7). doi: 10.1002/14651858.CD001871.pub4.
27. Buppasiri P, Lumbiganon P, Thinkhamrop J, Ngamjarus C, Laopaiboon M, Medley N. Calcium supplementation (other than for preventing or treating hypertension) for improving pregnancy and infant outcomes. Cochrane Database of Systematic Reviews. 2015 (2). doi: 10.1002/14651858.CD007079.pub3.
28. Carducci B, Keats E, Bhutta Z. Zinc supplementation for improving pregnancy and infant outcome. Cochrane Database of Systematic Reviews. 2021 2021(3). doi: doi:10.1002/14651858.CD000230.pub6.
29. Centeno Tablante E, Pachón H, Guetterman HM, Finkelstein JL. Fortification of wheat and maize flour with folic acid for population health outcomes. Cochrane Database of Systematic Reviews. 2019 (7). doi: 10.1002/14651858.CD012150.pub2.
30. Chastin S, Gardiner PA, Harvey JA, Leask CF, Jerez-Roig J, Rosenberg D, et al. Interventions for reducing sedentary behaviour in community‐dwelling older adults. Cochrane Database of Systematic Reviews. 2021 (6). doi: 10.1002/14651858.CD012784.pub2.
31. Chekima K, Yan S, Lee SH, Wong T, Noor M, Ooi Y, et al. Low glycaemic index or low glycaemic load diets for people with overweight or obesity. Cochrane Database of Systematic Reviews. 2023 2023(6). doi: doi:10.1002/14651858.CD005105.pub3.
32. Chen H, Zhuo Q, Yuan W, Wang J, Wu T. Vitamin A for preventing acute lower respiratory tract infections in children up to seven years of age. Cochrane Database of Systematic Reviews. 2008 2008(1). doi: doi:10.1002/14651858.CD006090.pub2.
33. Chen N, Yang M, Zhou M, Xiao J, Guo J, He L. L‐carnitine for cognitive enhancement in people without cognitive impairment. Cochrane Database of Systematic Reviews. 2017 (3). doi: 10.1002/14651858.CD009374.pub3.
34. Choi BKL, Verbeek JH, Tam WWS, Jiang JY. Exercises for prevention of recurrences of low‐back pain. Cochrane Database of Systematic Reviews. 2010 (1). doi: 10.1002/14651858.CD006555.pub2.
35. Clar C, Al-Khudairy L, Loveman E, Kelly SAM, Hartley L, Flowers N, et al. Low glycaemic index diets for the prevention of cardiovascular disease. Cochrane Database of Systematic Reviews. 2017 (7). doi: 10.1002/14651858.CD004467.pub3.
36. Colquitt J, Loveman E, O'Malley C, Azevedo L, Mead E, Al‐Khudairy L, et al. Diet, physical activity, and behavioural interventions for the treatment of overweight or obesity in preschool children up to the age of 6 years. Cochrane Database of Systematic Reviews. 2016 2016(3). doi: doi:10.1002/14651858.CD012105.
37. Cooper T, Teng C, Howell M, Teixeira-Pinto A, Jaure A, Wong G. D‐mannose for preventing and treating urinary tract infections. Cochrane Database of Systematic Reviews. 2022 2022(8). doi: doi:10.1002/14651858.CD013608.pub2.
38. Cormick G, Ciapponi A, Cafferata ML, Cormick MS, Belizán JM. Calcium supplementation for prevention of primary hypertension. Cochrane Database of Systematic Reviews. 2022 (1). doi: 10.1002/14651858.CD010037.pub4.
39. Cormick G, Ciapponi A, Harbron J, Perez SM, Vazquez P, Rivo J, et al. Calcium supplementation for people with overweight or obesity. Cochrane Database of Systematic Reviews. 2024 (5). doi: 10.1002/14651858.CD012268.pub2.
40. Crockett RA, King SE, Marteau TM, Prevost AT, Bignardi G, Roberts NW, et al. Nutritional labelling for healthier food or non‐alcoholic drink purchasing and consumption. Cochrane Database of Systematic Reviews. 2018 (2). doi: 10.1002/14651858.CD009315.pub2.
41. da Silva Lopes K, Yamaji N, Rahman M, Suto M, Takemoto Y, Garcia-Casal M, et al. Nutrition‐specific interventions for preventing and controlling anaemia throughout the life cycle: an overview of systematic reviews. Cochrane Database of Systematic Reviews. 2021 2021(9). doi: doi:10.1002/14651858.CD013092.pub2.
42. Dangour AD, Watson L, Cumming O, Boisson S, Che Y, Velleman Y, et al. Interventions to improve water quality and supply, sanitation and hygiene practices, and their effects on the nutritional status of children. Cochrane Database of Systematic Reviews. 2013 (8). doi: 10.1002/14651858.CD009382.pub2.
43. Das JK, Hoodbhoy Z, Salam RA, Bhutta AZ, Valenzuela‐Rubio NG, Weise Prinzo Z, et al. Lipid‐based nutrient supplements for maternal, birth, and infant developmental outcomes. Cochrane Database of Systematic Reviews. 2018 (8). doi: 10.1002/14651858.CD012610.pub2.
44. Das JK, Salam RA, Hadi YB, Sadiq Sheikh S, Bhutta AZ, Weise Prinzo Z, et al. Preventive lipid‐based nutrient supplements given with complementary foods to infants and young children 6 to 23 months of age for health, nutrition, and developmental outcomes. Cochrane Database of Systematic Reviews. 2019 (5). doi: 10.1002/14651858.CD012611.pub3.
45. Das JK, Salam RA, Mahmood SB, Moin A, Kumar R, Mukhtar K, et al. Food fortification with multiple micronutrients: impact on health outcomes in general population. Cochrane Database of Systematic Reviews. 2019 (12). doi: 10.1002/14651858.CD011400.pub2.
46. Davidson SJ, Barrett HL, Price SA, Callaway LK, Dekker Nitert M. Probiotics for preventing gestational diabetes. Cochrane Database of Systematic Reviews. 2021 (4). doi: 10.1002/14651858.CD009951.pub3.
47. De‐Regil LM, Jefferds MED, Peña‐Rosas JP. Point‐of‐use fortification of foods with micronutrient powders containing iron in children of preschool and school‐age. Cochrane Database of Systematic Reviews. 2017 (11). doi: 10.1002/14651858.CD009666.pub2.
48. De‐Regil LM, Jefferds MED, Sylvetsky AC, Dowswell T. Intermittent iron supplementation for improving nutrition and development in children under 12 years of age. Cochrane Database of Systematic Reviews. 2011 (12). doi: 10.1002/14651858.CD009085.pub2.
49. De‐Regil LM, Peña‐Rosas JP, Fernández‐Gaxiola AC, Rayco‐Solon P. Effects and safety of periconceptional oral folate supplementation for preventing birth defects. Cochrane Database of Systematic Reviews. 2015 (12). doi: 10.1002/14651858.CD007950.pub3.
50. Delgado‐Noguera MF, Calvache JA, Bonfill Cosp X, Kotanidou EP, Galli‐Tsinopoulou A. Supplementation with long chain polyunsaturated fatty acids (LCPUFA) to breastfeeding mothers for improving child growth and development. Cochrane Database of Systematic Reviews. 2015 (7). doi: 10.1002/14651858.CD007901.pub3.
51. Dickinson H, Bain E, Wilkinson D, Middleton P, Crowther C, Walker D. Creatine for women in pregnancy for neuroprotection of the fetus. Cochrane Database of Systematic Reviews. 2014 2014(12). doi: doi:10.1002/14651858.CD010846.pub2.
52. Duley L, Henderson‐Smart D, Meher S. Altered dietary salt for preventing pre‐eclampsia, and its complications. Cochrane Database of Systematic Reviews. 2005 2005(4). doi: doi:10.1002/14651858.CD005548.
53. Duley L, Henderson‐Smart DJ. Reduced salt intake compared to normal dietary salt, or high intake, in pregnancy. Cochrane Database of Systematic Reviews. 1999 (3). doi: 10.1002/14651858.CD001687.
54. Durao S, Visser ME, Ramokolo V, Oliveira JM, Schmidt BM, Balakrishna Y, et al. Community‐level interventions for improving access to food in low‐ and middle‐income countries. Cochrane Database of Systematic Reviews. 2020 (8). doi: 10.1002/14651858.CD011504.pub3.
55. Eaton JC, Rothpletz‐Puglia P, Dreker MR, Iannotti L, Lutter C, Kaganda J, et al. Effectiveness of provision of animal‐source foods for supporting optimal growth and development in children 6 to 59 months of age. Cochrane Database of Systematic Reviews. 2019 (2). doi: 10.1002/14651858.CD012818.pub2.
56. Ebrahim S, Taylor F, Ward K, Beswick A, Burke M, Davey Smith G. Multiple risk factor interventions for primary prevention of coronary heart disease. Cochrane Database of Systematic Reviews. 2011 2011(1). doi: doi:10.1002/14651858.CD001561.pub3.
57. Evans J, Lawrenson J. Antioxidant vitamin and mineral supplements for preventing age‐related macular degeneration. Cochrane Database of Systematic Reviews. 2017 2017(7). doi: doi:10.1002/14651858.CD000253.pub4.
58. Fair FJ, Ford GL, Soltani H. Interventions for supporting the initiation and continuation of breastfeeding among women who are overweight or obese. Cochrane Database of Systematic Reviews. 2019 (9). doi: 10.1002/14651858.CD012099.pub2.
59. Fallon A, Van der Putten D, Dring C, Moylett EH, Fealy G, Devane D. Baby‐led compared with scheduled (or mixed) breastfeeding for successful breastfeeding. Cochrane Database of Systematic Reviews. 2016 (9). doi: 10.1002/14651858.CD009067.pub3.
60. Field M, Mithra P, Peña-Rosas J. Wheat flour fortification with iron and other micronutrients for reducing anaemia and improving iron status in populations. Cochrane Database of Systematic Reviews. 2021 2021(1). doi: doi:10.1002/14651858.CD011302.pub3.
61. Filippini T, Malavolti M, Borrelli F, Izzo AA, Fairweather-Tait SJ, Horneber M, et al. Green tea (Camellia sinensis) for the prevention of cancer. Cochrane Database of Systematic Reviews. 2020 (3). doi: 10.1002/14651858.CD005004.pub3.
62. Finkelstein JL, Fothergill A, Venkatramanan S, Layden AJ, Williams JL, Crider KS, et al. Vitamin B12 supplementation during pregnancy for maternal and child health outcomes. Cochrane Database of Systematic Reviews. 2024 (1). doi: 10.1002/14651858.CD013823.pub2.
63. Flodgren G, Gonçalves‐Bradley D, Summerbell C. Interventions to change the behaviour of health professionals and the organisation of care to promote weight reduction in children and adults with overweight or obesity. Cochrane Database of Systematic Reviews. 2017 2017(11). doi: doi:10.1002/14651858.CD000984.pub3.
64. Flowers N, Hartley L, Todkill D, Stranges S, Rees K. Co‐enzyme Q10 supplementation for the primary prevention of cardiovascular disease. Cochrane Database of Systematic Reviews. 2014 (12). doi: 10.1002/14651858.CD010405.pub2.
65. Foong S, Tan M, Foong W, Marasco L, Ho J, Ong J. Oral galactagogues (natural therapies or drugs) for increasing breast milk production in mothers of non‐hospitalised term infants. Cochrane Database of Systematic Reviews. 2020 2020(5). doi: doi:10.1002/14651858.CD011505.pub2.
66. Foster C, Hillsdon M, Thorogood M, Kaur A, Wedatilake T. Interventions for promoting physical activity. Cochrane Database of Systematic Reviews. 2005 (1). doi: 10.1002/14651858.CD003180.pub2.
67. Foster C, Richards J, Thorogood M, Hillsdon M. Remote and web 2.0 interventions for promoting physical activity. Cochrane Database of Systematic Reviews. 2013 (9). doi: 10.1002/14651858.CD010395.pub2.
68. Freak-Poli RLA, Cumpston M, Albarqouni L, Clemes SA, Peeters A. Workplace pedometer interventions for increasing physical activity. Cochrane Database of Systematic Reviews. 2020 (7). doi: 10.1002/14651858.CD009209.pub3.
69. Furber CM, McGowan L, Bower P, Kontopantelis E, Quenby S, Lavender T. Antenatal interventions for reducing weight in obese women for improving pregnancy outcome. Cochrane Database of Systematic Reviews. 2013 (1). doi: 10.1002/14651858.CD009334.pub2.
70. Garcia‐Casal M, Peña‐Rosas J, De‐Regil L, Gwirtz J, Pasricha S. Fortification of maize flour with iron for controlling anaemia and iron deficiency in populations. Cochrane Database of Systematic Reviews. 2018 2018(12). doi: doi:10.1002/14651858.CD010187.pub2.
71. Gavine A, Shinwell S, Buchanan P, Farre A, Wade A, Lynn F, et al. Support for healthy breastfeeding mothers with healthy term babies. Cochrane Database of Systematic Reviews. 2022 2022(10). doi: doi:10.1002/14651858.CD001141.pub6.
72. Gillespie L, Robertson M, Gillespie W, Sherrington C, Gates S, Clemson L, et al. Interventions for preventing falls in older people living in the community. Cochrane Database of Systematic Reviews. 2012 2012(9). doi: doi:10.1002/14651858.CD007146.pub3.
73. Gogia S, Sachdev HS. Zinc supplementation for mental and motor development in children. Cochrane Database of Systematic Reviews. 2012 (12). doi: 10.1002/14651858.CD007991.pub2.
74. Goldenberg J, Yap C, Lytvyn L, Lo C, Beardsley J, Mertz D, et al. Probiotics for the prevention of Clostridium difficile‐associated diarrhea in adults and children. Cochrane Database of Systematic Reviews. 2017 2017(12). doi: doi:10.1002/14651858.CD006095.pub4.
75. Gonzalez-Garay AG, Serralde-Zúñiga AE, Medina Vera I, Velasco Hidalgo L, Alonso Ocaña MV. Higher versus lower protein intake in formula‐fed term infants. Cochrane Database of Systematic Reviews. 2023 (11). doi: 10.1002/14651858.CD013758.pub2.
76. Goudet S, Bogin B, Madise N, Griffiths P. Nutritional interventions for preventing stunting in children (birth to 59 months) living in urban slums in low‐ and middle‐income countries (LMIC). Cochrane Database of Systematic Reviews. 2019 2019(6). doi: doi:10.1002/14651858.CD011695.pub2.
77. Grande A, Keogh J, Silva V, Scott A. Exercise versus no exercise for the occurrence, severity, and duration of acute respiratory infections. Cochrane Database of Systematic Reviews. 2020 2020(4). doi: doi:10.1002/14651858.CD010596.pub3.
78. Graudal NA, Hubeck-Graudal T, Jurgens G. Effects of low sodium diet versus high sodium diet on blood pressure, renin, aldosterone, catecholamines, cholesterol, and triglyceride. Cochrane Database of Systematic Reviews. 2020 (12). doi: 10.1002/14651858.CD004022.pub5.
79. Griffith RJ, Alsweiler J, Moore AE, Brown S, Middleton P, Shepherd E, et al. Interventions to prevent women from developing gestational diabetes mellitus: an overview of Cochrane Reviews. Cochrane Database of Systematic Reviews. 2020 (6). doi: 10.1002/14651858.CD012394.pub3.
80. Gulani A, Sachdev HS. Zinc supplements for preventing otitis media. Cochrane Database of Systematic Reviews. 2014 (6). doi: 10.1002/14651858.CD006639.pub4.
81. Gunaratne AW, Makrides M, Collins CT. Maternal prenatal and/or postnatal n‐3 long chain polyunsaturated fatty acids (LCPUFA) supplementation for preventing allergies in early childhood. Cochrane Database of Systematic Reviews. 2015 (7). doi: 10.1002/14651858.CD010085.pub2.
82. Haider BA, Sharma R, Bhutta ZA. Neonatal vitamin A supplementation for the prevention of mortality and morbidity in term neonates in low and middle income countries. Cochrane Database of Systematic Reviews. 2017 (2). doi: 10.1002/14651858.CD006980.pub3.
83. Han S, Middleton P, Crowther CA. Exercise for pregnant women for preventing gestational diabetes mellitus. Cochrane Database of Systematic Reviews. 2012 (7). doi: 10.1002/14651858.CD009021.pub2.
84. Harding KB, Peña‐Rosas JP, Webster AC, Yap CMY, Payne BA, Ota E, et al. Iodine supplementation for women during the preconception, pregnancy and postpartum period. Cochrane Database of Systematic Reviews. 2017 (3). doi: 10.1002/14651858.CD011761.pub2.
85. Harris R, Gamboa A, Dailey Y, Ashcroft A. One‐to‐one dietary interventions undertaken in a dental setting to change dietary behaviour. Cochrane Database of Systematic Reviews. 2012 (3). doi: 10.1002/14651858.CD006540.pub2.
86. Hartley L, Clar C, Ghannam O, Flowers N, Stranges S, Rees K. Vitamin K for the primary prevention of cardiovascular disease. Cochrane Database of Systematic Reviews. 2015 (9). doi: 10.1002/14651858.CD011148.pub2.
87. Hartley L, Dyakova M, Holmes J, Clarke A, Lee MS, Ernst E, et al. Yoga for the primary prevention of cardiovascular disease. Cochrane Database of Systematic Reviews. 2014 (5). doi: 10.1002/14651858.CD010072.pub2.
88. Hartley L, Flowers N, Holmes J, Clarke A, Stranges S, Hooper L, et al. Green and black tea for the primary prevention of cardiovascular disease. Cochrane Database of Systematic Reviews. 2013 (6). doi: 10.1002/14651858.CD009934.pub2.
89. Hartley L, Flowers N, Lee MS, Ernst E, Rees K. Tai chi for primary prevention of cardiovascular disease. Cochrane Database of Systematic Reviews. 2014 (4). doi: 10.1002/14651858.CD010366.pub2.
90. Hartley L, Igbinedion E, Holmes J, Flowers N, Thorogood M, Clarke A, et al. Increased consumption of fruit and vegetables for the primary prevention of cardiovascular diseases. Cochrane Database of Systematic Reviews. 2013 (6). doi: 10.1002/14651858.CD009874.pub2.
91. Hartley L, Lee MS, Kwong JSW, Flowers N, Todkill D, Ernst E, et al. Qigong for the primary prevention of cardiovascular disease. Cochrane Database of Systematic Reviews. 2015 (6). doi: 10.1002/14651858.CD010390.pub2.
92. Hartley L, May MD, Loveman E, Colquitt JL, Rees K. Dietary fibre for the primary prevention of cardiovascular disease. Cochrane Database of Systematic Reviews. 2016 (1). doi: 10.1002/14651858.CD011472.pub2.
93. He F, Li J, MacGregor G. Effect of longer‐term modest salt reduction on blood pressure. Cochrane Database of Systematic Reviews. 2013 2013(4). doi: doi:10.1002/14651858.CD004937.pub2.
94. Hemilä H, Chalker E. Vitamin C for preventing and treating the common cold. Cochrane Database of Systematic Reviews. 2013 (1). doi: 10.1002/14651858.CD000980.pub4.
95. Hemilä H, Koivula T. Vitamin C for preventing and treating tetanus. Cochrane Database of Systematic Reviews. 2013 (11). doi: 10.1002/14651858.CD006665.pub3.
96. Hemilä H, Louhiala P. Vitamin C for preventing and treating pneumonia. Cochrane Database of Systematic Reviews. 2013 2013(8). doi: doi:10.1002/14651858.CD005532.pub3.
97. Hemmingsen B, Gimenez‐Perez G, Mauricio D, Roqué i Figuls M, Metzendorf MI, Richter B. Diet, physical activity or both for prevention or delay of type 2 diabetes mellitus and its associated complications in people at increased risk of developing type 2 diabetes mellitus. Cochrane Database of Systematic Reviews. 2017 (12). doi: 10.1002/14651858.CD003054.pub4.
98. Hodder RK, O'Brien KM, Tzelepis F, Wyse RJ, Wolfenden L. Interventions for increasing fruit and vegetable consumption in children aged five years and under. Cochrane Database of Systematic Reviews. 2020 (5). doi: 10.1002/14651858.CD008552.pub7.
99. Hofmeyr G, Lawrie T, Atallah Á, Torloni M. Calcium supplementation during pregnancy for preventing hypertensive disorders and related problems. Cochrane Database of Systematic Reviews. 2018 2018(10). doi: doi:10.1002/14651858.CD001059.pub5.
100. Hofmeyr GJ, Manyame S, Medley N, Williams MJ. Calcium supplementation commencing before or early in pregnancy, for preventing hypertensive disorders of pregnancy. Cochrane Database of Systematic Reviews. 2019 (9). doi: 10.1002/14651858.CD011192.pub3.
101. Hollands GJ, Carter P, Anwer S, King SE, Jebb SA, Ogilvie D, et al. Altering the availability or proximity of food, alcohol, and tobacco products to change their selection and consumption. Cochrane Database of Systematic Reviews. 2019 (9). doi: 10.1002/14651858.CD012573.pub3.
102. Hollands GJ, Shemilt I, Marteau TM, Jebb SA, Lewis HB, Wei Y, et al. Portion, package or tableware size for changing selection and consumption of food, alcohol and tobacco. Cochrane Database of Systematic Reviews. 2015 (9). doi: 10.1002/14651858.CD011045.pub2.
103. Hombali A, Solon J, Venkatesh B, Nair N, Peña‐Rosas J. Fortification of staple foods with vitamin A for vitamin A deficiency. Cochrane Database of Systematic Reviews. 2019 2019(5). doi: doi:10.1002/14651858.CD010068.pub2.
104. Hooper L, Al‐Khudairy L, Abdelhamid AS, Rees K, Brainard JS, Brown TJ, et al. Omega‐6 fats for the primary and secondary prevention of cardiovascular disease. Cochrane Database of Systematic Reviews. 2018 (11). doi: 10.1002/14651858.CD011094.pub4.
105. Hooper L, Bartlett C, Davey Smith G, Ebrahim S. Advice to reduce dietary salt for prevention of cardiovascular disease. Cochrane Database of Systematic Reviews. 2004 (1). doi: 10.1002/14651858.CD003656.pub2.
106. Hooper L, Martin N, Jimoh OF, Kirk C, Foster E, Abdelhamid AS. Reduction in saturated fat intake for cardiovascular disease. Cochrane Database of Systematic Reviews. 2020 (8). doi: 10.1002/14651858.CD011737.pub3.
107. Hooper L, Summerbell CD, Thompson R, Sills D, Roberts FG, Moore HJ, et al. Reduced or modified dietary fat for preventing cardiovascular disease. Cochrane Database of Systematic Reviews. 2012 (5). doi: 10.1002/14651858.CD002137.pub3.
108. Howe T, Shea B, Dawson L, Downie F, Murray A, Ross C, et al. Exercise for preventing and treating osteoporosis in postmenopausal women. Cochrane Database of Systematic Reviews. 2011 2011(7). doi: doi:10.1002/14651858.CD000333.pub2.
109. Howe TE, Rochester L, Neil F, Skelton DA, Ballinger C. Exercise for improving balance in older people. Cochrane Database of Systematic Reviews. 2011 (11). doi: 10.1002/14651858.CD004963.pub3.
110. Huey SL, Acharya N, Silver A, Sheni R, Yu EA, Peña-Rosas JP, et al. Effects of oral vitamin D supplementation on linear growth and other health outcomes among children under five years of age. Cochrane Database of Systematic Reviews. 2020 (12). doi: 10.1002/14651858.CD012875.pub2.
111. Iheozor‐Ejiofor Z, Worthington HV, Walsh T, O'Malley L, Clarkson JE, Macey R, et al. Water fluoridation for the prevention of dental caries. Cochrane Database of Systematic Reviews. 2015 (6). doi: 10.1002/14651858.CD010856.pub2.
112. Imdad A, Ahmed Z, Bhutta ZA. Vitamin A supplementation for the prevention of morbidity and mortality in infants one to six months of age. Cochrane Database of Systematic Reviews. 2016 (9). doi: 10.1002/14651858.CD007480.pub3.
113. Imdad A, Mayo-Wilson E, Haykal MR, Regan A, Sidhu J, Smith A, et al. Vitamin A supplementation for preventing morbidity and mortality in children from six months to five years of age. Cochrane Database of Systematic Reviews. 2022 (3). doi: 10.1002/14651858.CD008524.pub4.
114. Imdad A, Rogner J, Sherwani R, Sidhu J, Regan A, Haykal M, et al. Zinc supplementation for preventing mortality, morbidity, and growth failure in children aged 6 months to 12 years. Cochrane Database of Systematic Reviews. 2023 2023(3). doi: doi:10.1002/14651858.CD009384.pub3.
115. Jahanfar S, Jaafar SH. Effects of restricted caffeine intake by mother on fetal, neonatal and pregnancy outcomes. Cochrane Database of Systematic Reviews. 2015 (6). doi: 10.1002/14651858.CD006965.pub4.
116. Jalal CSB, De-Regil LM, Pike V, Mithra P. Fortification of condiments and seasonings with iron for preventing anaemia and improving health. Cochrane Database of Systematic Reviews. 2023 (9). doi: 10.1002/14651858.CD009604.pub2.
117. Jasani B, Simmer K, Patole S, Rao S. Long chain polyunsaturated fatty acid supplementation in infants born at term. Cochrane Database of Systematic Reviews. 2017 2017(3). doi: doi:10.1002/14651858.CD000376.pub4.
118. Jin H, Leng Q, Li C. Dietary flavonoid for preventing colorectal neoplasms. Cochrane Database of Systematic Reviews. 2012 2012(8). doi: doi:10.1002/14651858.CD009350.pub2.
119. Jull AB, Ni Mhurchu C, Bennett DA, Dunshea‐Mooij CAE, Rodgers A. Chitosan for overweight or obesity. Cochrane Database of Systematic Reviews. 2008 (3). doi: 10.1002/14651858.CD003892.pub3.
120. Jurgens TM, Whelan AM, Killian L, Doucette S, Kirk S, Foy E. Green tea for weight loss and weight maintenance in overweight or obese adults. Cochrane Database of Systematic Reviews. 2012 (12). doi: 10.1002/14651858.CD008650.pub2.
121. Keats EC, Haider BA, Tam E, Bhutta ZA. Multiple‐micronutrient supplementation for women during pregnancy. Cochrane Database of Systematic Reviews. 2019 (3). doi: 10.1002/14651858.CD004905.pub6.
122. Kelly SAM, Hartley L, Loveman E, Colquitt JL, Jones HM, Al-Khudairy L, et al. Whole grain cereals for the primary or secondary prevention of cardiovascular disease. Cochrane Database of Systematic Reviews. 2017 (8). doi: 10.1002/14651858.CD005051.pub3.
123. Kendrick D, Kumar A, Carpenter H, Zijlstra GAR, Skelton DA, Cook JR, et al. Exercise for reducing fear of falling in older people living in the community. Cochrane Database of Systematic Reviews. 2014 (11). doi: 10.1002/14651858.CD009848.pub2.
124. Kramer M, Kakuma R. Maternal dietary antigen avoidance during pregnancy or lactation, or both, for preventing or treating atopic disease in the child. Cochrane Database of Systematic Reviews. 2012 2012(9). doi: doi:10.1002/14651858.CD000133.pub3.
125. Kramer M, Kakuma R. Optimal duration of exclusive breastfeeding. Cochrane Database of Systematic Reviews. 2012 2012(8). doi: doi:10.1002/14651858.CD003517.pub2.
126. Kramer MS, McDonald SW. Aerobic exercise for women during pregnancy. Cochrane Database of Systematic Reviews. 2006 (3). doi: 10.1002/14651858.CD000180.pub2.
127. Kristjansson B, Petticrew M, MacDonald B, Krasevec J, Janzen L, Greenhalgh T, et al. School feeding for improving the physical and psychosocial health of disadvantaged students. Cochrane Database of Systematic Reviews. 2007 2007(1). doi: doi:10.1002/14651858.CD004676.pub2.
128. Kristjansson E, Francis D, Liberato S, Benkhalti Jandu M, Welch V, Batal M, et al. Food supplementation for improving the physical and psychosocial health of socio‐economically disadvantaged children aged three months to five years. Cochrane Database of Systematic Reviews. 2015 2015(3). doi: doi:10.1002/14651858.CD009924.pub2.
129. Langford R, Bonell CP, Jones HE, Pouliou T, Murphy SM, Waters E, et al. The WHO Health Promoting School framework for improving the health and well‐being of students and their academic achievement. Cochrane Database of Systematic Reviews. 2014 (4). doi: 10.1002/14651858.CD008958.pub2.
130. Larun L, Nordheim LV, Ekeland E, Hagen KB, Heian F. Exercise in prevention and treatment of anxiety and depression among children and young people. Cochrane Database of Systematic Reviews. 2006 (3). doi: 10.1002/14651858.CD004691.pub2.
131. Lassi ZS, Kurji J, Oliveira CSD, Moin A, Bhutta ZA. Zinc supplementation for the promotion of growth and prevention of infections in infants less than six months of age. Cochrane Database of Systematic Reviews. 2020 (4). doi: 10.1002/14651858.CD010205.pub2.
132. Lassi ZS, Moin A, Bhutta ZA. Zinc supplementation for the prevention of pneumonia in children aged 2 months to 59 months. Cochrane Database of Systematic Reviews. 2016 (12). doi: 10.1002/14651858.CD005978.pub3.
133. Lassi ZS, Salam RA, Haider BA, Bhutta ZA. Folic acid supplementation during pregnancy for maternal health and pregnancy outcomes. Cochrane Database of Systematic Reviews. 2013 (3). doi: 10.1002/14651858.CD006896.pub2.
134. Lawrenson J, Evans J. Omega 3 fatty acids for preventing or slowing the progression of age‐related macular degeneration. Cochrane Database of Systematic Reviews. 2015 2015(4). doi: doi:10.1002/14651858.CD010015.pub3.
135. Lazzerini M, Rubert L, Pani P. Specially formulated foods for treating children with moderate acute malnutrition in low‐ and middle‐income countries. Cochrane Database of Systematic Reviews. 2013 2013(6). doi: doi:10.1002/14651858.CD009584.pub2.
136. Lerch C, Meissner T. Interventions for the prevention of nutritional rickets in term born children. Cochrane Database of Systematic Reviews. 2007 2007(4). doi: doi:10.1002/14651858.CD006164.pub2.
137. Lhachimi SK, Pega F, Heise TL, Fenton C, Gartlehner G, Griebler U, et al. Taxation of the fat content of foods for reducing their consumption and preventing obesity or other adverse health outcomes. Cochrane Database of Systematic Reviews. 2020 (9). doi: 10.1002/14651858.CD012415.pub2.
138. Liddle S, Pennick V. Interventions for preventing and treating low‐back and pelvic pain during pregnancy. Cochrane Database of Systematic Reviews. 2015 2015(9). doi: doi:10.1002/14651858.CD001139.pub4.
139. Liu CJ, Latham NK. Progressive resistance strength training for improving physical function in older adults. Cochrane Database of Systematic Reviews. 2009 (3). doi: 10.1002/14651858.CD002759.pub2.
140. Loveman E, Al‐Khudairy L, Johnson R, Robertson W, Colquitt J, Mead E, et al. Parent‐only interventions for childhood overweight or obesity in children aged 5 to 11 years. Cochrane Database of Systematic Reviews. 2015 2015(12). doi: doi:10.1002/14651858.CD012008.
141. Low MSY, Speedy J, Styles CE, De‐Regil LM, Pasricha SR. Daily iron supplementation for improving anaemia, iron status and health in menstruating women. Cochrane Database of Systematic Reviews. 2016 (4). doi: 10.1002/14651858.CD009747.pub2.
142. Lumbiganon P, Martis R, Laopaiboon M, Festin M, Ho J, Hakimi M. Antenatal breastfeeding education for increasing breastfeeding duration. Cochrane Database of Systematic Reviews. 2016 2016(12). doi: doi:10.1002/14651858.CD006425.pub4.
143. Maas T, Kaper J, Sheikh A, Knottnerus J, Wesseling G, Dompeling E, et al. Mono and multifaceted inhalant and/or food allergen reduction interventions for preventing asthma in children at high risk of developing asthma. Cochrane Database of Systematic Reviews. 2009 2009(3). doi: doi:10.1002/14651858.CD006480.pub2.
144. Makrides M, Crosby DD, Shepherd E, Crowther CA. Magnesium supplementation in pregnancy. Cochrane Database of Systematic Reviews. 2014 (4). doi: 10.1002/14651858.CD000937.pub2.
145. Malouf R, Grimley Evans J. Folic acid with or without vitamin B12 for the prevention and treatment of healthy elderly and demented people. Cochrane Database of Systematic Reviews. 2008 2008(4). doi: doi:10.1002/14651858.CD004514.pub2.
146. Martin A, Booth JN, Laird Y, Sproule J, Reilly JJ, Saunders DH. Physical activity, diet and other behavioural interventions for improving cognition and school achievement in children and adolescents with obesity or overweight. Cochrane Database of Systematic Reviews. 2018 (3). doi: 10.1002/14651858.CD009728.pub4.
147. Martin N, Germanò R, Hartley L, Adler AJ, Rees K. Nut consumption for the primary prevention of cardiovascular disease. Cochrane Database of Systematic Reviews. 2015 (9). doi: 10.1002/14651858.CD011583.pub2.
148. Mathew M, Ervin A, Tao J, Davis R. Antioxidant vitamin supplementation for preventing and slowing the progression of age‐related cataract. Cochrane Database of Systematic Reviews. 2012 2012(6). doi: doi:10.1002/14651858.CD004567.pub2.
149. McCauley ME, van den Broek N, Dou L, Othman M. Vitamin A supplementation during pregnancy for maternal and newborn outcomes. Cochrane Database of Systematic Reviews. 2015 (10). doi: 10.1002/14651858.CD008666.pub3.
150. McCleery J, Abraham R, Denton D, Rutjes A, Chong L, Al‐Assaf A, et al. Vitamin and mineral supplementation for preventing dementia or delaying cognitive decline in people with mild cognitive impairment. Cochrane Database of Systematic Reviews. 2018 2018(11). doi: doi:10.1002/14651858.CD011905.pub2.
151. McLaren L, Sumar N, Barberio AM, Trieu K, Lorenzetti DL, Tarasuk V, et al. Population‐level interventions in government jurisdictions for dietary sodium reduction. Cochrane Database of Systematic Reviews. 2016 (9). doi: 10.1002/14651858.CD010166.pub2.
152. Mead E, Brown T, Rees K, Azevedo L, Whittaker V, Jones D, et al. Diet, physical activity and behavioural interventions for the treatment of overweight or obese children from the age of 6 to 11 years. Cochrane Database of Systematic Reviews. 2017 2017(6). doi: doi:10.1002/14651858.CD012651.
153. Meher S, Duley L. Exercise or other physical activity for preventing pre‐eclampsia and its complications. Cochrane Database of Systematic Reviews. 2006 (2). doi: 10.1002/14651858.CD005942.
154. Meher S, Duley L. Garlic for preventing pre‐eclampsia and its complications. Cochrane Database of Systematic Reviews. 2006 (3). doi: 10.1002/14651858.CD006065.
155. Metzendorf M-I, Wieland L, Richter B. Mobile health (m‐health) smartphone interventions for adolescents and adults with overweight or obesity. Cochrane Database of Systematic Reviews. 2024 2024(2). doi: doi:10.1002/14651858.CD013591.pub2.
156. Middleton P, Gomersall JC, Gould JF, Shepherd E, Olsen SF, Makrides M. Omega‐3 fatty acid addition during pregnancy. Cochrane Database of Systematic Reviews. 2018 (11). doi: 10.1002/14651858.CD003402.pub3.
157. Miller BJ, Murray L, Beckmann MM, Kent T, Macfarlane B. Dietary supplements for preventing postnatal depression. Cochrane Database of Systematic Reviews. 2013 (10). doi: 10.1002/14651858.CD009104.pub2.
158. Milne A, Potter J, Vivanti A, Avenell A. Protein and energy supplementation in elderly people at risk from malnutrition. Cochrane Database of Systematic Reviews. 2009 2009(2). doi: doi:10.1002/14651858.CD003288.pub3.
159. Montgomery P, Dennis J. Physical exercise for sleep problems in adults aged 60+. Cochrane Database of Systematic Reviews. 2002 2002(4). doi: doi:10.1002/14651858.CD003404.
160. Morgan E, Schoonees A, Sriram U, Faure M, Seguin‐Fowler R. Caregiver involvement in interventions for improving children's dietary intake and physical activity behaviors. Cochrane Database of Systematic Reviews. 2020 2020(1). doi: doi:10.1002/14651858.CD012547.pub2.
161. Mosdøl A, Lidal IB, Straumann GH, Vist GE. Targeted mass media interventions promoting healthy behaviours to reduce risk of non‐communicable diseases in adult, ethnic minorities. Cochrane Database of Systematic Reviews. 2017 (2). doi: 10.1002/14651858.CD011683.pub2.
162. Motuhifonua S, Lin L, Alsweiler J, Crawford T, Crowther C. Antenatal dietary supplementation with myo‐inositol for preventing gestational diabetes. Cochrane Database of Systematic Reviews. 2023 2023(2). doi: doi:10.1002/14651858.CD011507.pub3.
163. Muktabhant B, Lawrie TA, Lumbiganon P, Laopaiboon M. Diet or exercise, or both, for preventing excessive weight gain in pregnancy. Cochrane Database of Systematic Reviews. 2015 (6). doi: 10.1002/14651858.CD007145.pub3.
164. Murtagh EM, Murphy MH, Milton K, Roberts NW, O'Gorman CSM, Foster C. Interventions outside the workplace for reducing sedentary behaviour in adults under 60 years of age. Cochrane Database of Systematic Reviews. 2020 (7). doi: 10.1002/14651858.CD012554.pub2.
165. Naude CE, Brand A, Schoonees A, Nguyen KA, Chaplin M, Volmink J. Low‐carbohydrate versus balanced‐carbohydrate diets for reducing weight and cardiovascular risk. Cochrane Database of Systematic Reviews. 2022 (1). doi: 10.1002/14651858.CD013334.pub2.
166. Naude CE, Visser ME, Nguyen KA, Durao S, Schoonees A. Effects of total fat intake on bodyweight in children. Cochrane Database of Systematic Reviews. 2018 (7). doi: 10.1002/14651858.CD012960.pub2.
167. Ndikom C, Fawole B, Ilesanmi R. Extra fluids for breastfeeding mothers for increasing milk production. Cochrane Database of Systematic Reviews. 2014 2014(6). doi: doi:10.1002/14651858.CD008758.pub2.
168. Neil-Sztramko SE, Caldwell H, Dobbins M. School‐based physical activity programs for promoting physical activity and fitness in children and adolescents aged 6 to 18. Cochrane Database of Systematic Reviews. 2021 (9). doi: 10.1002/14651858.CD007651.pub3.
169. Neuberger A, Okebe J, Yahav D, Paul M. Oral iron supplements for children in malaria‐endemic areas. Cochrane Database of Systematic Reviews. 2016 (2). doi: 10.1002/14651858.CD006589.pub4.
170. Odigwe CC, Smedslund G, Ejemot‐Nwadiaro RI, Anyanechi CC, Krawinkel MB. Supplementary vitamin E, selenium, cysteine and riboflavin for preventing kwashiorkor in preschool children in developing countries. Cochrane Database of Systematic Reviews. 2010 (4). doi: 10.1002/14651858.CD008147.pub2.
171. Ojha S, Elfzzani Z, Kwok TC, Dorling J. Education of family members to support weaning to solids and nutrition in later infancy in term‐born infants. Cochrane Database of Systematic Reviews. 2020 (7). doi: 10.1002/14651858.CD012241.pub2.
172. Oliveira JM, Allert R, East CE. Vitamin A supplementation for postpartum women. Cochrane Database of Systematic Reviews. 2016 (3). doi: 10.1002/14651858.CD005944.pub3.
173. Ong TG, Gordon M, Banks SSC, Thomas MR, Akobeng AK. Probiotics to prevent infantile colic. Cochrane Database of Systematic Reviews. 2019 (3). doi: 10.1002/14651858.CD012473.pub2.
174. Ooi C, Loke S, Yassin Z, Hamid T. Carbohydrates for improving the cognitive performance of independent‐living older adults with normal cognition or mild cognitive impairment. Cochrane Database of Systematic Reviews. 2011 2011(4). doi: doi:10.1002/14651858.CD007220.pub2.
175. Osborn D, Sinn J. Soy formula for prevention of allergy and food intolerance in infants. Cochrane Database of Systematic Reviews. 2006 2006(4). doi: doi:10.1002/14651858.CD003741.pub4.
176. Osborn D, Sinn J. Probiotics in infants for prevention of allergic disease and food hypersensitivity. Cochrane Database of Systematic Reviews. 2007 2007(4). doi: doi:10.1002/14651858.CD006475.pub2.
177. Osborn D, Sinn J. Prebiotics in infants for prevention of allergy. Cochrane Database of Systematic Reviews. 2013 2013(3). doi: doi:10.1002/14651858.CD006474.pub3.
178. Osborn D, Sinn J, Jones L. Infant formulas containing hydrolysed protein for prevention of allergic disease. Cochrane Database of Systematic Reviews. 2018 2018(10). doi: doi:10.1002/14651858.CD003664.pub6.
179. Ota E, da Silva Lopes K, Middleton P, Flenady V, Wariki W, Rahman M, et al. Antenatal interventions for preventing stillbirth, fetal loss and perinatal death: an overview of Cochrane systematic reviews. Cochrane Database of Systematic Reviews. 2020 2020(12). doi: doi:10.1002/14651858.CD009599.pub2.
180. Ota E, Hori H, Mori R, Tobe‐Gai R, Farrar D. Antenatal dietary education and supplementation to increase energy and protein intake. Cochrane Database of Systematic Reviews. 2015 2015(6). doi: doi:10.1002/14651858.CD000032.pub3.
181. Padhani Z, Moazzam Z, Ashraf A, Bilal H, Salam R, Das J, et al. Vitamin C supplementation for prevention and treatment of pneumonia. Cochrane Database of Systematic Reviews. 2021 2021(11). doi: doi:10.1002/14651858.CD013134.pub3.
182. Palacios C, Kostiuk LK, Peña‐Rosas JP. Vitamin D supplementation for women during pregnancy. Cochrane Database of Systematic Reviews. 2019 (7). doi: 10.1002/14651858.CD008873.pub4.
183. Palmer M, Henschke N, Bergman H, Villanueva G, Maayan N, Tamrat T, et al. Targeted client communication via mobile devices for improving maternal, neonatal, and child health. Cochrane Database of Systematic Reviews. 2020 2020(8). doi: doi:10.1002/14651858.CD013679.
184. Parry SP, Coenen P, Shrestha N, O'Sullivan PB, Maher CG, Straker LM. Workplace interventions for increasing standing or walking for decreasing musculoskeletal symptoms in sedentary workers. Cochrane Database of Systematic Reviews. 2019 (11). doi: 10.1002/14651858.CD012487.pub2.
185. Peña‐Rosas J, Mithra P, Unnikrishnan B, Kumar N, De‐Regil L, Nair N, et al. Fortification of rice with vitamins and minerals for addressing micronutrient malnutrition. Cochrane Database of Systematic Reviews. 2019 2019(10). doi: doi:10.1002/14651858.CD009902.pub2.
186. Peña‐Rosas JP, De‐Regil LM, Garcia‐Casal MN, Dowswell T. Daily oral iron supplementation during pregnancy. Cochrane Database of Systematic Reviews. 2015 (7). doi: 10.1002/14651858.CD004736.pub5.
187. Peña‐Rosas JP, De‐Regil LM, Gomez Malave H, Flores‐Urrutia MC, Dowswell T. Intermittent oral iron supplementation during pregnancy. Cochrane Database of Systematic Reviews. 2015 (10). doi: 10.1002/14651858.CD009997.pub2.
188. Petkovic J, Duench S, Trawin J, Dewidar O, Pardo Pardo J, Simeon R, et al. Behavioural interventions delivered through interactive social media for health behaviour change, health outcomes, and health equity in the adult population. Cochrane Database of Systematic Reviews. 2021 2021(5). doi: doi:10.1002/14651858.CD012932.pub2.
189. Pfinder M, Heise TL, Hilton Boon M, Pega F, Fenton C, Griebler U, et al. Taxation of unprocessed sugar or sugar‐added foods for reducing their consumption and preventing obesity or other adverse health outcomes. Cochrane Database of Systematic Reviews. 2020 (4). doi: 10.1002/14651858.CD012333.pub2.
190. Priebe M, van Binsbergen J, de Vos R, Vonk RJ. Whole grain foods for the prevention of type 2 diabetes mellitus. Cochrane Database of Systematic Reviews. 2008 (1). doi: 10.1002/14651858.CD006061.pub2.
191. Priest N, Armstrong R, Doyle J, Waters E. Policy interventions implemented through sporting organisations for promoting healthy behaviour change. Cochrane Database of Systematic Reviews. 2008 (3). doi: 10.1002/14651858.CD004809.pub3.
192. Priest N, Armstrong R, Doyle J, Waters E. Interventions implemented through sporting organisations for increasing participation in sport. Cochrane Database of Systematic Reviews. 2008 2008(3). doi: doi:10.1002/14651858.CD004812.pub3.
193. Rees K, Al-Khudairy L, Takeda A, Stranges S. Vegan dietary pattern for the primary and secondary prevention of cardiovascular diseases. Cochrane Database of Systematic Reviews. 2021 (2). doi: 10.1002/14651858.CD013501.pub2.
194. Rees K, Dyakova M, Wilson N, Ward K, Thorogood M, Brunner E. Dietary advice for reducing cardiovascular risk. Cochrane Database of Systematic Reviews. 2013 (12). doi: 10.1002/14651858.CD002128.pub5.
195. Rees K, Hartley L, Day C, Flowers N, Clarke A, Stranges S. Selenium supplementation for the primary prevention of cardiovascular disease. Cochrane Database of Systematic Reviews. 2013 2013(1). doi: doi:10.1002/14651858.CD009671.pub2.
196. Rees K, Takeda A, Martin N, Ellis L, Wijesekara D, Vepa A, et al. Mediterranean‐style diet for the primary and secondary prevention of cardiovascular disease. Cochrane Database of Systematic Reviews. 2019 (3). doi: 10.1002/14651858.CD009825.pub3.
197. Richards J, Hillsdon M, Thorogood M, Foster C. Face‐to‐face interventions for promoting physical activity. Cochrane Database of Systematic Reviews. 2013 (9). doi: 10.1002/14651858.CD010392.pub2.
198. Richards J, Thorogood M, Hillsdon M, Foster C. Face‐to‐face versus remote and web 2.0 interventions for promoting physical activity. Cochrane Database of Systematic Reviews. 2013 (9). doi: 10.1002/14651858.CD010393.pub2.
199. Riggs E, Kilpatrick N, Slack‐Smith L, Chadwick B, Yelland J, Muthu M, et al. Interventions with pregnant women, new mothers and other primary caregivers for preventing early childhood caries. Cochrane Database of Systematic Reviews. 2019 2019(11). doi: doi:10.1002/14651858.CD012155.pub2.
200. Riley P, Moore D, Ahmed F, Sharif M, Worthington H. Xylitol‐containing products for preventing dental caries in children and adults. Cochrane Database of Systematic Reviews. 2015 2015(3). doi: doi:10.1002/14651858.CD010743.pub2.
201. Rumbold A, Ota E, Hori H, Miyazaki C, Crowther CA. Vitamin E supplementation in pregnancy. Cochrane Database of Systematic Reviews. 2015 (9). doi: 10.1002/14651858.CD004069.pub3.
202. Rumbold A, Ota E, Nagata C, Shahrook S, Crowther CA. Vitamin C supplementation in pregnancy. Cochrane Database of Systematic Reviews. 2015 (9). doi: 10.1002/14651858.CD004072.pub3.
203. Rutjes AWS, Denton DA, Di Nisio M, Chong LY, Abraham RP, Al‐Assaf AS, et al. Vitamin and mineral supplementation for maintaining cognitive function in cognitively healthy people in mid and late life. Cochrane Database of Systematic Reviews. 2018 (12). doi: 10.1002/14651858.CD011906.pub2.
204. Salam RA, Zuberi NF, Bhutta ZA. Pyridoxine (vitamin B6) supplementation during pregnancy or labour for maternal and neonatal outcomes. Cochrane Database of Systematic Reviews. 2015 (6). doi: 10.1002/14651858.CD000179.pub3.
205. Santos J, Christoforou A, Trieu K, McKenzie B, Downs S, Billot L, et al. Iodine fortification of foods and condiments, other than salt, for preventing iodine deficiency disorders. Cochrane Database of Systematic Reviews. 2019 2019(2). doi: doi:10.1002/14651858.CD010734.pub2.
206. Schindler T, Sinn J, Osborn D. Polyunsaturated fatty acid supplementation in infancy for the prevention of allergy. Cochrane Database of Systematic Reviews. 2016 2016(10). doi: doi:10.1002/14651858.CD010112.pub2.
207. Schmucker C, Eisele-Metzger A, Meerpohl JJ, Lehane C, Kuellenberg de Gaudry D, Lohner S, et al. Effects of a gluten‐reduced or gluten‐free diet for the primary prevention of cardiovascular disease. Cochrane Database of Systematic Reviews. 2022 (2). doi: 10.1002/14651858.CD013556.pub2.
208. Schwenger EM, Tejani AM, Loewen PS. Probiotics for preventing urinary tract infections in adults and children. Cochrane Database of Systematic Reviews. 2015 (12). doi: 10.1002/14651858.CD008772.pub2.
209. Scott AM, Clark J, Julien B, Islam F, Roos K, Grimwood K, et al. Probiotics for preventing acute otitis media in children. Cochrane Database of Systematic Reviews. 2019 (6). doi: 10.1002/14651858.CD012941.pub2.
210. Seron P, Lanas F, Pardo Hernandez H, Bonfill Cosp X. Exercise for people with high cardiovascular risk. Cochrane Database of Systematic Reviews. 2014 (8). doi: 10.1002/14651858.CD009387.pub2.
211. Sguassero Y, de Onis M, Bonotti AM, Carroli G. Community‐based supplementary feeding for promoting the growth of children under five years of age in low and middle income countries. Cochrane Database of Systematic Reviews. 2012 (6). doi: 10.1002/14651858.CD005039.pub3.
212. Shah D, Sachdev HS, Gera T, De‐Regil LM, Peña‐Rosas JP. Fortification of staple foods with zinc for improving zinc status and other health outcomes in the general population. Cochrane Database of Systematic Reviews. 2016 (6). doi: 10.1002/14651858.CD010697.pub2.
213. Shaw K, Gennat H, O'Rourke P, Del Mar C. Exercise for overweight or obesity. Cochrane Database of Systematic Reviews. 2006 2006(4). doi: doi:10.1002/14651858.CD003817.pub3.
214. Shepherd E, Gomersall JC, Tieu J, Han S, Crowther CA, Middleton P. Combined diet and exercise interventions for preventing gestational diabetes mellitus. Cochrane Database of Systematic Reviews. 2017 (11). doi: 10.1002/14651858.CD010443.pub3.
215. Sherrington C, Fairhall NJ, Wallbank GK, Tiedemann A, Michaleff ZA, Howard K, et al. Exercise for preventing falls in older people living in the community. Cochrane Database of Systematic Reviews. 2019 (1). doi: 10.1002/14651858.CD012424.pub2.
216. Showell M, Mackenzie-Proctor R, Jordan V, Hart R. Antioxidants for female subfertility. Cochrane Database of Systematic Reviews. 2020 2020(8). doi: doi:10.1002/14651858.CD007807.pub4.
217. Shrestha N, Kukkonen‐Harjula KT, Verbeek JH, Ijaz S, Hermans V, Pedisic Z. Workplace interventions for reducing sitting at work. Cochrane Database of Systematic Reviews. 2018 (12). doi: 10.1002/14651858.CD010912.pub5.
218. Smith HA, Becker GE. Early additional food and fluids for healthy breastfed full‐term infants. Cochrane Database of Systematic Reviews. 2016 (8). doi: 10.1002/14651858.CD006462.pub4.
219. Suchdev PS, Jefferds MED, Ota E, da Silva Lopes K, De‐Regil LM. Home fortification of foods with multiple micronutrient powders for health and nutrition in children under two years of age. Cochrane Database of Systematic Reviews. 2020 (2). doi: 10.1002/14651858.CD008959.pub3.
220. Suchdev PS, Peña‐Rosas JP, De‐Regil LM. Multiple micronutrient powders for home (point‐of‐use) fortification of foods in pregnant women. Cochrane Database of Systematic Reviews. 2015 (6). doi: 10.1002/14651858.CD011158.pub2.
221. Sydenham E, Dangour AD, Lim WS. Omega 3 fatty acid for the prevention of cognitive decline and dementia. Cochrane Database of Systematic Reviews. 2012 (6). doi: 10.1002/14651858.CD005379.pub3.
222. Takahashi R, Ota E, Hoshi K, Naito T, Toyoshima Y, Yuasa H, et al. Fluoride supplementation (with tablets, drops, lozenges or chewing gum) in pregnant women for preventing dental caries in the primary teeth of their children. Cochrane Database of Systematic Reviews. 2017 (10). doi: 10.1002/14651858.CD011850.pub2.
223. Tan ML, Abrams SA, Osborn DA. Vitamin D supplementation for term breastfed infants to prevent vitamin D deficiency and improve bone health. Cochrane Database of Systematic Reviews. 2020 (12). doi: 10.1002/14651858.CD013046.pub2.
224. Thompson RL, Summerbell CD, Hooper L, Higgins JPT, Little P, Talbot D, et al. Dietary advice given by a dietitian versus other health professional or self‐help resources to reduce blood cholesterol. Cochrane Database of Systematic Reviews. 2003 (3). doi: 10.1002/14651858.CD001366.
225. Tieu J, Shepherd E, Middleton P, Crowther CA. Dietary advice interventions in pregnancy for preventing gestational diabetes mellitus. Cochrane Database of Systematic Reviews. 2017 (1). doi: 10.1002/14651858.CD006674.pub3.
226. Tubert‐Jeannin S, Auclair C, Amsallem E, Tramini P, Gerbaud L, Ruffieux C, et al. Fluoride supplements (tablets, drops, lozenges or chewing gums) for preventing dental caries in children. Cochrane Database of Systematic Reviews. 2011 (12). doi: 10.1002/14651858.CD007592.pub2.
227. Turawa E, Musekiwa A, Rohwer A. Interventions for preventing postpartum constipation. Cochrane Database of Systematic Reviews. 2020 2020(8). doi: doi:10.1002/14651858.CD011625.pub3.
228. Uthman O, Hartley L, Rees K, Taylor F, Ebrahim S, Clarke A. Multiple risk factor interventions for primary prevention of cardiovascular disease in low‐ and middle‐income countries. Cochrane Database of Systematic Reviews. 2015 2015(8). doi: doi:10.1002/14651858.CD011163.pub2.
229. Valentín‐Gudiol M, Mattern‐Baxter K, Girabent‐Farrés M, Bagur‐Calafat C, Hadders‐Algra M, Angulo‐Barroso R. Treadmill interventions in children under six years of age at risk of neuromotor delay. Cochrane Database of Systematic Reviews. 2017 2017(7). doi: doi:10.1002/14651858.CD009242.pub3.
230. Verhagen A, Bierma‐Zeinstra S, Burdorf A, Stynes S, de Vet H, Koes B. Conservative interventions for treating work‐related complaints of the arm, neck or shoulder in adults. Cochrane Database of Systematic Reviews. 2013 2013(12). doi: doi:10.1002/14651858.CD008742.pub2.
231. Vinceti M, Filippini T, Del Giovane C, Dennert G, Zwahlen M, Brinkman M, et al. Selenium for preventing cancer. Cochrane Database of Systematic Reviews. 2018 (1). doi: 10.1002/14651858.CD005195.pub4.
232. Virgara R, Phillips A, Lewis LK, Baldock K, Wolfenden L, Ferguson T, et al. Interventions in outside‐school hours childcare settings for promoting physical activity amongst schoolchildren aged 4 to 12 years. Cochrane Database of Systematic Reviews. 2021 (9). doi: 10.1002/14651858.CD013380.pub2.
233. Visser J, McLachlan M, Maayan N, Garner P. Community‐based supplementary feeding for food insecure, vulnerable and malnourished populations – an overview of systematic reviews. Cochrane Database of Systematic Reviews. 2018 2018(11). doi: doi:10.1002/14651858.CD010578.pub2.
234. Visser ME, Schoonees A, Ezekiel CN, Randall NP, Naude CE. Agricultural and nutritional education interventions for reducing aflatoxin exposure to improve infant and child growth in low‐ and middle‐income countries. Cochrane Database of Systematic Reviews. 2020 (4). doi: 10.1002/14651858.CD013376.pub2.
235. von Philipsborn P, Stratil JM, Burns J, Busert LK, Pfadenhauer LM, Polus S, et al. Environmental interventions to reduce the consumption of sugar‐sweetened beverages and their effects on health. Cochrane Database of Systematic Reviews. 2019 (6). doi: 10.1002/14651858.CD012292.pub2.
236. Weingarten M, Zalmanovici Trestioreanu A, Yaphe J. Dietary calcium supplementation for preventing colorectal cancer and adenomatous polyps. Cochrane Database of Systematic Reviews. 2008 2008(1). doi: doi:10.1002/14651858.CD003548.pub4.
237. Wieland L, Falzon L, Sciamanna C, Trudeau K, Brodney Folse S, Schwartz J, et al. Interactive computer‐based interventions for weight loss or weight maintenance in overweight or obese people. Cochrane Database of Systematic Reviews. 2012 2012(8). doi: doi:10.1002/14651858.CD007675.pub2.
238. Wieland L, Skoetz N, Pilkington K, Harbin S, Vempati R, Berman B. Yoga for chronic non‐specific low back pain. Cochrane Database of Systematic Reviews. 2022 2022(11). doi: doi:10.1002/14651858.CD010671.pub3.
239. Winzenberg TM, Powell S, Shaw KA, Jones G. Vitamin D supplementation for improving bone mineral density in children. Cochrane Database of Systematic Reviews. 2010 (10). doi: 10.1002/14651858.CD006944.pub2.
240. Winzenberg TM, Shaw KA, Fryer J, Jones G. Calcium supplementation for improving bone mineral density in children. Cochrane Database of Systematic Reviews. 2006 (2). doi: 10.1002/14651858.CD005119.pub2.
241. Wolfenden L, Barnes C, Jones J, Finch M, Wyse RJ, Kingsland M, et al. Strategies to improve the implementation of healthy eating, physical activity and obesity prevention policies, practices or programmes within childcare services. Cochrane Database of Systematic Reviews. 2020 (2). doi: 10.1002/14651858.CD011779.pub3.
242. Wolfenden L, McCrabb S, Barnes C, O'Brien KM, Ng KW, Nathan NK, et al. Strategies for enhancing the implementation of school‐based policies or practices targeting diet, physical activity, obesity, tobacco or alcohol use. Cochrane Database of Systematic Reviews. 2022 (8). doi: 10.1002/14651858.CD011677.pub3.
243. Wu T, Liu G, Li P, Clar C. Iodised salt for preventing iodine deficiency disorders. Cochrane Database of Systematic Reviews. 2002 2002(3). doi: doi:10.1002/14651858.CD003204.
244. Yakoob MY, Salam RA, Khan FR, Bhutta ZA. Vitamin D supplementation for preventing infections in children under five years of age. Cochrane Database of Systematic Reviews. 2016 (11). doi: 10.1002/14651858.CD008824.pub2.
245. Yamato T, Maher C, Saragiotto B, Hancock M, Ostelo R, Cabral C, et al. Pilates for low back pain. Cochrane Database of Systematic Reviews. 2015 2015(7). doi: doi:10.1002/14651858.CD010265.pub2.
246. Yeung CA, Chong LY, Glenny AM. Fluoridated milk for preventing dental caries. Cochrane Database of Systematic Reviews. 2015 (9). doi: 10.1002/14651858.CD003876.pub4.
247. Yoong SL, Lum M, Wolfenden L, Jackson J, Barnes C, Hall AE, et al. Healthy eating interventions delivered in early childhood education and care settings for improving the diet of children aged six months to six years. Cochrane Database of Systematic Reviews. 2023 (8). doi: 10.1002/14651858.CD013862.pub3.
248. Young J, Angevaren M, Rusted J, Tabet N. Aerobic exercise to improve cognitive function in older people without known cognitive impairment. Cochrane Database of Systematic Reviews. 2015 (4). doi: 10.1002/14651858.CD005381.pub4.
249. Zhao Y, Dong BR, Hao Q. Probiotics for preventing acute upper respiratory tract infections. Cochrane Database of Systematic Reviews. 2022 (8). doi: 10.1002/14651858.CD006895.pub4.
