# Supplementary material for: Dissemination patterns of Cochrane reviews on nutrition and physical activity using Altmetric data: a bibliographic study
Source: Syst Rev. 2026 Feb 23;15:104. doi: 10.1186/s13643-026-03127-8 (PMC13037133; doi:10.1186/s13643-026-03127-8)
Supplement: Supplementary file 3 — Supplementary Material 3. Sensitivity analysis. [file 13643_2026_3127_MOESM3_ESM.docx]

Appendix 3

Sensitivity analysis

Contents

[Medians 2](#_Toc215561148)

[Outliers 3](#_Toc215561149)

[Descriptive statistics 13](#_Toc215561150)

[Multicollinearity 17](#_Toc215561151)

[Sensitivity analysis 1: Linear regression Altmetric Scores 18](#_Toc215561152)

[Sensitivity analysis 2: Linear regression Dimensions Citations Scores 20](#_Toc215561153)

# Medians

| **Statistics** | | | | | | | |
| --- | --- | --- | --- | --- | --- | --- | --- |
|  | | Altmetric score | Dimensions score | Publication year | Included studies | Meta-analysis: yes vs. no (R) | PLS languages number |
| N | Valid | 249 | 249 | 249 | 249 | 249 | 249 |
|  | Missing | 0 | 0 | 0 | 0 | 0 | 0 |
| Mean | | 125.68 | 150.91 | 2015.58 | 24.69 | .80 | 9.26 |
| Median | | 48.00 | 81.00 | 2016.00 | 13.00 | 1.00 | 10.00 |
| Minimum | | 3 | 0 | 1999 | 0 | 0 | 2 |
| Maximum | | 4111 | 2700 | 2024 | 195 | 1 | 17 |

# Outliers

**Altmetric score**

Altmetric score Stem-and-Leaf Plot

Frequency Stem & Leaf

58.00 0 . 0000000000000000001111111111111111111111111111111111111111

54.00 0 . 222222222222222222222222222222222233333333333333333333

31.00 0 . 4444444444444444444555555555555

17.00 0 . 66666666677777777

16.00 0 . 8888888888899999

16.00 1 . 0000000011111111

2.00 1 . 23

6.00 1 . 455555

5.00 1 . 66777

4.00 1 . 8999

8.00 2 . 00000111

3.00 2 . 333

29.00 Extremes (>=253)

Stem width: 100

Each leaf: 1 case(s)


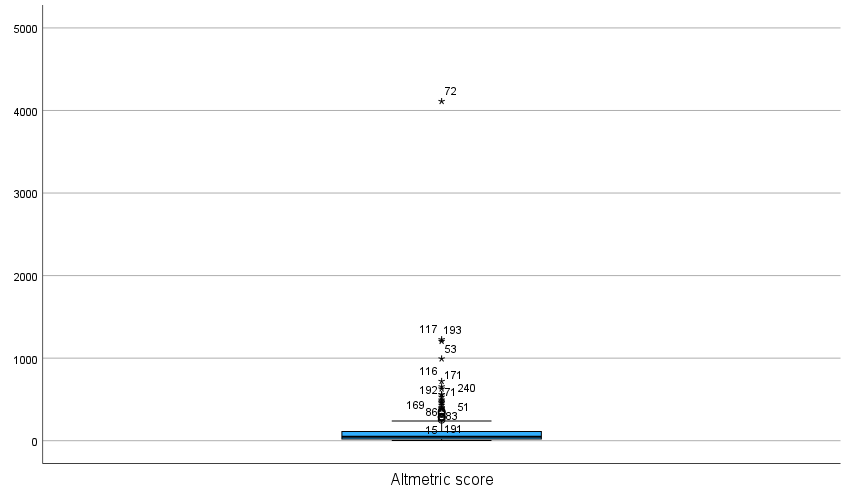


**Dimensions score**

Dimensions score Stem-and-Leaf Plot

Frequency Stem & Leaf

25.00 0 . 0000000000111111111111111

30.00 0 . 222222222222233333333333333333

33.00 0 . 444444444444445555555555555555555

33.00 0 . 666666666666677777777777777777777

16.00 0 . 8888888888999999

15.00 1 . 000000000111111

17.00 1 . 22222222222233333

18.00 1 . 444444444445555555

5.00 1 . 66777

9.00 1 . 889999999

4.00 2 . 1111

3.00 2 . 233

3.00 2 . 455

4.00 2 . 6677

3.00 2 . 999

5.00 3 . 00011

1.00 3 . 2

25.00 Extremes (>=347)

Stem width: 100

Each leaf: 1 case(s)


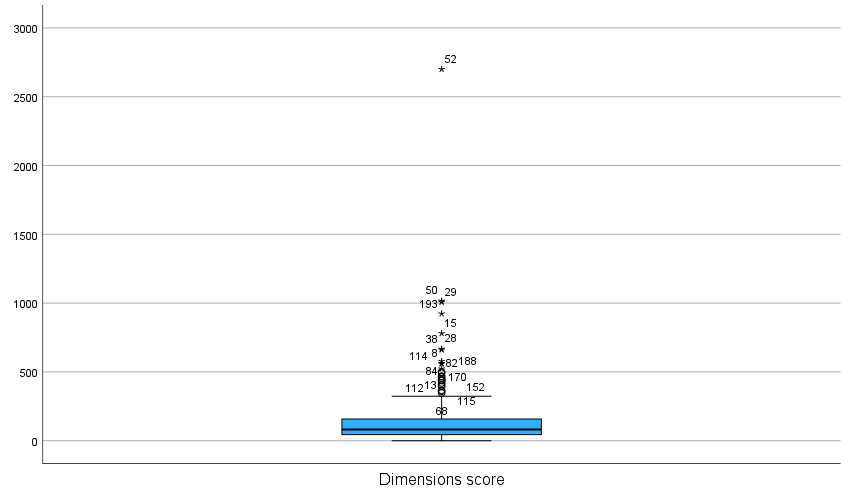


**Publication year**

Publication year Stem-and-Leaf Plot

Frequency Stem & Leaf

4.00 Extremes (=<2003)

.00 200 .

4.00 200 . 4555

10.00 200 . 6666666777

11.00 200 . 88888888999

10.00 201 . 0001111111

33.00 201 . 222222222222223333333333333333333

45.00 201 . 444444444444445555555555555555555555555555555

38.00 201 . 66666666666666666667777777777777777777

38.00 201 . 88888888888888889999999999999999999999

35.00 202 . 00000000000000000000000001111111111

18.00 202 . 222222222222333333

3.00 202 . 444

Stem width: 10

Each leaf: 1 case(s)


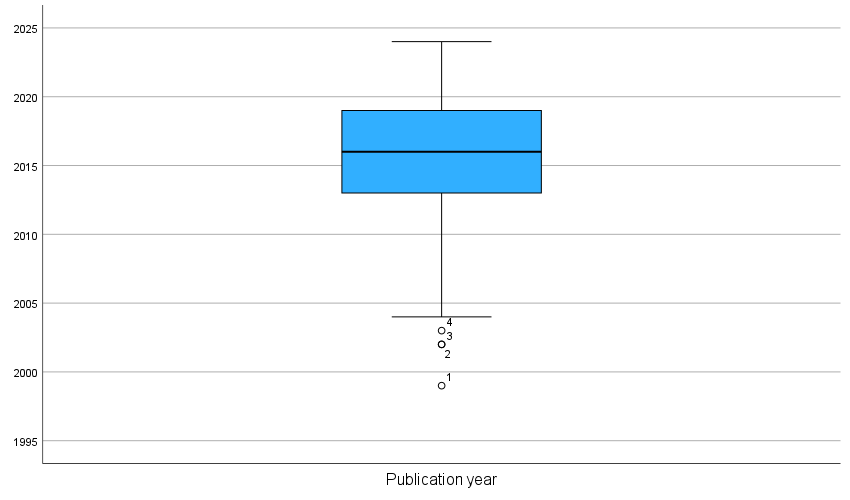


**Included studies**

Included studies Stem-and-Leaf Plot

Frequency Stem & Leaf

42.00 0 . 000000011111111111122222222222333334444444

44.00 0 . 55555555555566666666777777788888888888999999

48.00 1 . 000000000000111111111112222222222333333444444444

25.00 1 . 5555566677777788888899999

18.00 2 . 000111111133333444

12.00 2 . 556777888999

10.00 3 . 0001233444

3.00 3 . 589

10.00 4 . 0113333444

4.00 4 . 5789

2.00 5 . 23

3.00 5 . 589

3.00 6 . 112

25.00 Extremes (>=63)

Stem width: 10

Each leaf: 1 case(s)


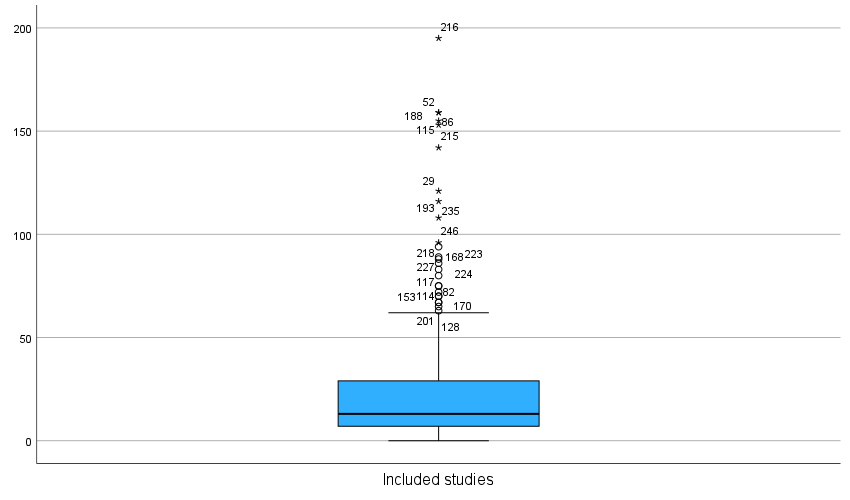


**PLS languages number**

PLS languages number Stem-and-Leaf Plot

Frequency Stem & Leaf

3.00 2 . 000

9.00 3 . 000000000

11.00 4 . 00000000000

14.00 5 . 00000000000000

14.00 6 . 00000000000000

19.00 7 . 0000000000000000000

26.00 8 . 00000000000000000000000000

28.00 9 . 0000000000000000000000000000

30.00 10 . 000000000000000000000000000000

27.00 11 . 000000000000000000000000000

31.00 12 . 0000000000000000000000000000000

16.00 13 . 0000000000000000

14.00 14 . 00000000000000

5.00 15 . 00000

1.00 16 . 0

1.00 17 . 0

Stem width: 1

Each leaf: 1 case(s)


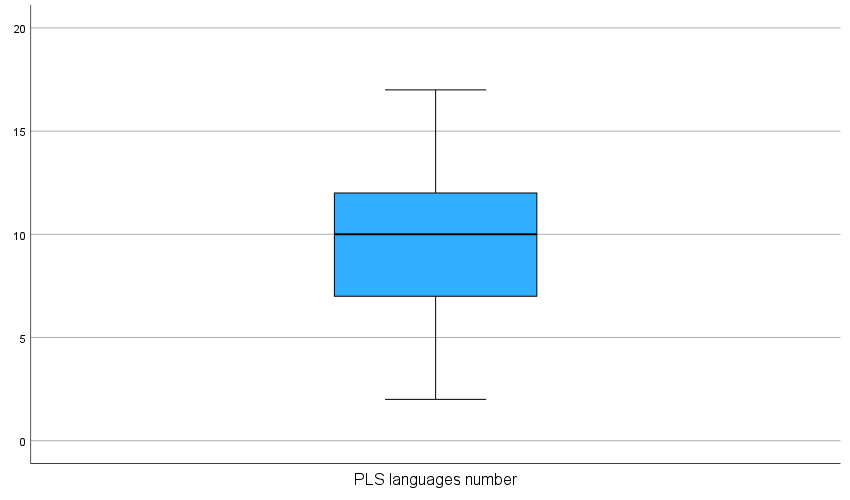


# Descriptive statistics

| **Review type** | | | | | |
| --- | --- | --- | --- | --- | --- |
|  | | Frequency | Percent | Valid Percent | Cumulative Percent |
| Valid | Overview | 4 | 1.6 | 1.6 | 1.6 |
|  | Systematic Review | 245 | 98.4 | 98.4 | 100.0 |
|  | Total | 249 | 100.0 | 100.0 |  |

| **Altmetric interpretation** | | | | | |
| --- | --- | --- | --- | --- | --- |
|  | | Frequency | Percent | Valid Percent | Cumulative Percent |
| Valid | no interpretation | 5 | 2.0 | 2.0 | 2.0 |
|  | in less than 25% of traced outputs | 10 | 4.0 | 4.0 | 6.0 |
|  | in 25% of traced outputs | 86 | 34.5 | 34.5 | 40.6 |
|  | in 5% of traced outputs | 148 | 59.4 | 59.4 | 100.0 |
|  | Total | 249 | 100.0 | 100.0 |  |

| **Altmetric Score: high (49-4111) vs. low (3-48) (R)** | | | | | |
| --- | --- | --- | --- | --- | --- |
|  | | Frequency | Percent | Valid Percent | Cumulative Percent |
| Valid | lower Altmetric scores 3-48 | 126 | 50.6 | 50.6 | 50.6 |
|  | higher Altmetric scores 49-4111 | 123 | 49.4 | 49.4 | 100.0 |
|  | Total | 249 | 100.0 | 100.0 |  |

| **Dimensions Score: high (82-2700) vs. low (0-81) (R)** | | | | | |
| --- | --- | --- | --- | --- | --- |
|  | | Frequency | Percent | Valid Percent | Cumulative Percent |
| Valid | lower Dimensions scores (0-81) | 125 | 50.2 | 50.2 | 50.2 |
|  | higher Dimensions scores (82-2700) | 124 | 49.8 | 49.8 | 100.0 |
|  | Total | 249 | 100.0 | 100.0 |  |

| **Publication year: newer (2017-2024) vs. older (1999-2016) (R)** | | | | | |
| --- | --- | --- | --- | --- | --- |
|  | | Frequency | Percent | Valid Percent | Cumulative Percent |
| Valid | older (1999-2016) | 136 | 54.6 | 54.6 | 54.6 |
|  | newer (2017-2024) | 113 | 45.4 | 45.4 | 100.0 |
|  | Total | 249 | 100.0 | 100.0 |  |

| **Included studies: many (14-195) vs. few (0-13) (R)** | | | | | |
| --- | --- | --- | --- | --- | --- |
|  | | Frequency | Percent | Valid Percent | Cumulative Percent |
| Valid | 0-13 studies | 125 | 50.2 | 50.2 | 50.2 |
|  | 14-195 studies | 124 | 49.8 | 49.8 | 100.0 |
|  | Total | 249 | 100.0 | 100.0 |  |

| **Meta-analysis: yes vs. no (R)** | | | | | |
| --- | --- | --- | --- | --- | --- |
|  | | Frequency | Percent | Valid Percent | Cumulative Percent |
| Valid | no | 51 | 20.5 | 20.5 | 20.5 |
|  | yes | 198 | 79.5 | 79.5 | 100.0 |
|  | Total | 249 | 100.0 | 100.0 |  |

| **PLS languages number** | | | | | |
| --- | --- | --- | --- | --- | --- |
|  | | Frequency | Percent | Valid Percent | Cumulative Percent |
| Valid | 2 | 3 | 1.2 | 1.2 | 1.2 |
|  | 3 | 9 | 3.6 | 3.6 | 4.8 |
|  | 4 | 11 | 4.4 | 4.4 | 9.2 |
|  | 5 | 14 | 5.6 | 5.6 | 14.9 |
|  | 6 | 14 | 5.6 | 5.6 | 20.5 |
|  | 7 | 19 | 7.6 | 7.6 | 28.1 |
|  | 8 | 26 | 10.4 | 10.4 | 38.6 |
|  | 9 | 28 | 11.2 | 11.2 | 49.8 |
|  | 10 | 30 | 12.0 | 12.0 | 61.8 |
|  | 11 | 27 | 10.8 | 10.8 | 72.7 |
|  | 12 | 31 | 12.4 | 12.4 | 85.1 |
|  | 13 | 16 | 6.4 | 6.4 | 91.6 |
|  | 14 | 14 | 5.6 | 5.6 | 97.2 |
|  | 15 | 5 | 2.0 | 2.0 | 99.2 |
|  | 16 | 1 | .4 | .4 | 99.6 |
|  | 17 | 1 | .4 | .4 | 100.0 |
|  | Total | 249 | 100.0 | 100.0 |  |

# Multicollinearity

| **Correlations (highest correlation coefficients highlighted)** | | | | | | | |
| --- | --- | --- | --- | --- | --- | --- | --- |
|  | | Altmetric Score: high (49-4111) vs. low (3-48) (R) | Dimensions Score: high (82-2700) vs. low (0-81) (R) | Publication year: newer (2017-2024) vs. older (1999-2016) (R) | Included studies: many (14-195) vs. few (0-13) (R) | Meta-analysis: yes vs. no (R) | PLS languages number |
| Altmetric Score: high (49-4111) vs. low (3-48) (R) | Pearson Correlation | 1 | .430^**^ | .035 | .285^**^ | .183^**^ | .333^**^ |
|  | Sig. (2-tailed) |  | <.001 | .581 | <.001 | .004 | <.001 |
|  | N | 249 | 249 | 249 | 249 | 249 | 249 |
| Dimensions Score: high (82-2700) vs. low (0-81) (R) | Pearson Correlation | .430^**^ | 1 | -.295^**^ | .309^**^ | .326^**^ | -.053 |
|  | Sig. (2-tailed) | <.001 |  | <.001 | <.001 | <.001 | .406 |
|  | N | 249 | 249 | 249 | 249 | 249 | 249 |
| Publication year: newer (2017-2024) vs. older (1999-2016) (R) | Pearson Correlation | .035 | -.295^**^ | 1 | .205^**^ | .103 | .367^**^ |
|  | Sig. (2-tailed) | .581 | <.001 |  | .001 | .105 | <.001 |
|  | N | 249 | 249 | 249 | 249 | 249 | 249 |
| Included studies: many (14-195) vs. few (0-13) (R) | Pearson Correlation | .285^**^ | .309^**^ | .205^**^ | 1 | .386^**^ | .203^**^ |
|  | Sig. (2-tailed) | <.001 | <.001 | .001 |  | <.001 | .001 |
|  | N | 249 | 249 | 249 | 249 | 249 | 249 |
| Meta-analysis: yes vs. no (R) | Pearson Correlation | .183^**^ | .326^**^ | .103 | .386^**^ | 1 | .145^*^ |
|  | Sig. (2-tailed) | .004 | <.001 | .105 | <.001 |  | .022 |
|  | N | 249 | 249 | 249 | 249 | 249 | 249 |
| PLS languages number | Pearson Correlation | .333^**^ | -.053 | .367^**^ | .203^**^ | .145^*^ | 1 |
|  | Sig. (2-tailed) | <.001 | .406 | <.001 | .001 | .022 |  |
|  | N | 249 | 249 | 249 | 249 | 249 | 249 |
| **. Correlation is significant at the 0.01 level (2-tailed). | | | | | | | |
| *. Correlation is significant at the 0.05 level (2-tailed). | | | | | | | |

# Sensitivity analysis 1: Linear regression Altmetric Scores

Variables:

1 dependent variable: ranked Altmetric Scores

5 independent variables: ranked publication year, ranked studies included in review, meta-analysis conducted in review, PLS languages, and ranked Dimensions Citations Scores

| **Model Summary** | | | | |
| --- | --- | --- | --- | --- |
| Model | R | R Square | Adjusted R Square | Std. Error of the Estimate |
| 1 | .701^a^ | .491 | .480 | 51.909 |
| a. Predictors: (Constant), Rank of Dimensions_Score, PLS languages number, Meta-analysis: yes vs. no (R), Rank of Included_studies, Rank of Publication_year | | | | |

| **ANOVA^a^** | | | | | | |
| --- | --- | --- | --- | --- | --- | --- |
| Model | | Sum of Squares | df | Mean Square | F | Sig. |
| 1 | Regression | 631505.256 | 5 | 126301.051 | 46.872 | <.001^b^ |
|  | Residual | 654785.744 | 243 | 2694.592 |  |  |
|  | Total | 1286291.000 | 248 |  |  |  |
| a. Dependent Variable: Rank of Altmetric_Score | | | | | | |
| b. Predictors: (Constant), Rank of Dimensions_Score, PLS languages number, Meta-analysis: yes vs. no (R), Rank of Included_studies, Rank of Publication_year | | | | | | |

| **Coefficients^a^** | | | | | | | | |
| --- | --- | --- | --- | --- | --- | --- | --- | --- |
| Model | | Unstandardized Coefficients | | Standardized Coefficients | t | Sig. | 95.0% Confidence Interval for B | |
|  |  | B | Std. Error | Beta |  |  | Lower Bound | Upper Bound |
| 1 | (Constant) | -45.946 | 13.058 |  | -3.519 | <.001 | -71.666 | -20.225 |
|  | Rank of Publication_year | .217 | .074 | .216 | 2.918 | .004 | .070 | .363 |
|  | Rank of Included_studies | .035 | .062 | .035 | .555 | .579 | -.088 | .158 |
|  | Meta-analysis: yes vs. no (R) | -18.756 | 9.617 | -.105 | -1.950 | .052 | -37.700 | .187 |
|  | PLS languages number | 7.786 | 1.312 | .343 | 5.934 | <.001 | 5.202 | 10.370 |
|  | Rank of Dimensions_Score | .659 | .068 | .659 | 9.723 | <.001 | .526 | .793 |
| a. Dependent Variable: Rank of Altmetric_Score | | | | | | | | |

# Sensitivity analysis 2: Linear regression Dimensions Citations Scores

Variables:

1 dependent variable: ranked Dimensions Citations Scores

5 independent variables: ranked publication year, ranked studies included in review, meta-analysis conducted in review, PLS languages, and ranked Altmetric Scores

| **Model Summary** | | | | |
| --- | --- | --- | --- | --- |
| Model | R | R Square | Adjusted R Square | Std. Error of the Estimate |
| 1 | .820^a^ | .672 | .665 | 41.682 |
| a. Predictors: (Constant), Rank of Altmetric_Score, Rank of Publication_year, Meta-analysis: yes vs. no (R), Rank of Included_studies, PLS languages number | | | | |

| **ANOVA^a^** | | | | | | |
| --- | --- | --- | --- | --- | --- | --- |
| Model | | Sum of Squares | df | Mean Square | F | Sig. |
| 1 | Regression | 864206.751 | 5 | 172841.350 | 99.483 | <.001^b^ |
|  | Residual | 422186.249 | 243 | 1737.392 |  |  |
|  | Total | 1286393.000 | 248 |  |  |  |
| a. Dependent Variable: Rank of Dimensions_Score | | | | | | |
| b. Predictors: (Constant), Rank of Altmetric_Score, Rank of Publication_year, Meta-analysis: yes vs. no (R), Rank of Included_studies, PLS languages number | | | | | | |

| **Coefficients^a^** | | | | | | | | |
| --- | --- | --- | --- | --- | --- | --- | --- | --- |
| Model | | Unstandardized Coefficients | | Standardized Coefficients | t | Sig. | 95.0% Confidence Interval for B | |
|  |  | B | Std. Error | Beta |  |  | Lower Bound | Upper Bound |
| 1 | (Constant) | 86.023 | 9.224 |  | 9.326 | <.001 | 67.853 | 104.193 |
|  | Rank of Publication_year | -.599 | .047 | -.597 | -12.780 | <.001 | -.692 | -.507 |
|  | Rank of Included_studies | .313 | .046 | .313 | 6.810 | <.001 | .223 | .404 |
|  | Meta-analysis: yes vs. no (R) | 29.606 | 7.547 | .166 | 3.923 | <.001 | 14.739 | 44.472 |
|  | PLS languages number | -.210 | 1.127 | -.009 | -.186 | .852 | -2.430 | 2.010 |
|  | Rank of Altmetric_Score | .425 | .044 | .425 | 9.723 | <.001 | .339 | .511 |
| a. Dependent Variable: Rank of Dimensions_Score | | | | | | | | |
