# Supplementary material for: Dissemination patterns of Cochrane reviews on nutrition and physical activity using Altmetric data: a bibliographic study
Source: Syst Rev. 2026 Feb 23;15:104. doi: 10.1186/s13643-026-03127-8 (PMC13037133; doi:10.1186/s13643-026-03127-8)
Supplement: Supplementary file 4 — Supplementary Material 4. Plain language summary. [file 13643_2026_3127_MOESM4_ESM.docx]

**Appendix 4**

**Plain language summary (PLS)**

English:

This study looked at how much attention was given to 249 scientific papers. The papers were about diet and exercise. They were published in 1999-2024. Each had a short summary in simple language. These were written in 2-17 languages, especially English, Spanish and Arabic. People talked about these papers on social media, like Twitter/X, Facebook, and Wikipedia. Scientists also talked about these papers in scientific papers. People talked more about these papers online if scientists also talked about them in scientific papers and if they had summaries in many languages. Scientists talked more about these papers in scientific papers if people talked about them online, if they were older, and had more results and a specific analysis. The 249 scientific papers are talked about in social media and in scientific papers. But we do not know if medical workers use them in real life.

German:

In dieser Studie wurde untersucht, wie viel Aufmerksamkeit 249 wissenschaftliche Texte über Ernährung und Bewegung bekommen haben. Die Texte wurden zwischen 1999 und 2024 veröffentlicht. Sie hatten kurze Zusammenfassungen in einfacher Sprache. Diese Zusammenfassungen gab es in 2 bis 17 Sprachen. Besonders oft waren sie auf Englisch, Spanisch und Arabisch. Die Leute haben in sozialen Medien wie Twitter/X, Facebook und Wikipedia über diese Texte gesprochen. Forschende haben auch in wissenschaftlichen Texten über diese Texte berichtet. Die Leute sprachen online mehr über diese Texte, wenn Forschende über sie auch in wissenschaftlichen Texten berichteten und wenn sie Zusammenfassungen in einfacher Sprache in vielen Sprachen hatten. Forschende berichteten in wissenschaftlichen Texten mehr über diese Texte, wenn die Leute online über diese Texte sprachen, wenn die Texte älter waren und mehr Ergebnisse und eine spezifische Analyse enthielten. Die 249 wissenschaftliche Texte werden in sozialen Medien und in wissenschaftlichen Texten besprochen. Aber wir wissen nicht, ob medizinische Fachkräfte sie in der Praxis nutzen.
